# The application of Raman spectroscopy for the diagnosis and monitoring of lung tumors

**DOI:** 10.3389/fbioe.2024.1385552

**Published:** 2024-04-18

**Authors:** Yuyang Miao, Lihong Wu, Junlian Qiang, Jinfeng Qi, Ying Li, Ruihua Li, Xiaodong Kong, Qiang Zhang

**Affiliations:** Department of Geriatrics, Tianjin Medical University General Hospital, Tianjin Geriatrics Institute, Tianjin, China

**Keywords:** lung tumors, lung adenocarcinoma, lung squamous cell carcinoma, Raman spectroscopy, surface-enhanced Raman scattering

## Abstract

Raman spectroscopy is an optical technique that uses inelastic light scattering in response to vibrating molecules to produce chemical fingerprints of tissues, cells, and biofluids. Raman spectroscopy strategies produce high levels of chemical specificity without requiring extensive sample preparation, allowing for the use of advanced optical tools such as microscopes, fiber optics, and lasers that operate in the visible and near-infrared spectral range, making them increasingly suitable for a wide range of medical diagnostic applications. Metal nanoparticles and nonlinear optical effects can improve Raman signals, and optimized fiber optic Raman probes can make real-time, *in vivo,* single-point observations. Furthermore, diagnostic speed and spatial accuracy can be improved through the multimodal integration of Raman measurements and other technologies. Recent studies have significantly contributed to the improvement of diagnostic speed and accuracy, making them suitable for clinical application. Lung cancer is a prevalent type of respiratory malignancy. However, the use of computed tomography for detection and screening frequently reveals numerous smaller lung nodules, which makes the diagnostic process more challenging from a clinical perspective. While the majority of small nodules detected are benign, there are currently no direct methods for identifying which nodules represent very early-stage lung cancer. Positron emission tomography and other auxiliary diagnostic methods for non-surgical biopsy samples from these small nodules yield low detection rates, which might result in significant expenses and the possibility of complications for patients. While certain subsets of patients can undergo curative treatment, other individuals have a less favorable prognosis and need alternative therapeutic interventions. With the emergence of new methods for treating cancer, such as immunotherapies, which can potentially extend patient survival and even lead to a complete cure in certain instances, it is crucial to determine the most suitable biomarkers and metrics for assessing the effectiveness of these novel compounds. This will ensure that significant treatment outcomes are accurately measured. This review provides a comprehensive overview of the prospects of Raman spectroscopy and its applications in the diagnosis and analysis of lung tumors.

## Introduction

Benign lung tumors can develop in the lung parenchyma, bronchi, or visceral pleura. The occurrence of these benign tumors is infrequent and encompasses a variety of conditions such as pulmonary pseudotumors, bronchial leiomyomas, papillomas, hamartomas, lung lipomas, neurogenic tumors, lung fibromas, bronchial chondromas, and benign lung clear cell tumors. Various tumor-like lesions can also arise, such as those resulting from infections or congenital disorders, including lung cysts, pulmonary pseudotumors, hamartomas, cases of pulmonary pseudolymphoma, and pulmonary sclerosing hemangiomas.

Lung cancers, also known as primary bronchial lung cancers, are the most lethal malignant tumors worldwide. While low-dose computed tomography (CT) screening strategies can help in diagnosing lung cancer, there are now various methods being used for the staging and diagnosis of affected patients. Treatments provided to patients are specific to the stage and subtype of disease. A variety of personalized therapeutic regimens have been developed, making the management of lung cancer patients quite complex (Nasim et al., 2019). Significant advancements have been made in various areas of lung cancer control in recent years. These include a more comprehensive understanding of the causes of the disease, improved methods for early detection, diagnosis, and therapy, and better provision of end-of-life care for affected patients. This continuum of cancer control refers to the comprehensive application of different therapeutic strategies, such as immunotherapy, targeted therapy, radiotherapy, chemotherapy, and surgery to control tumor growth and maintain a state of stable or gradual disease progression over long periods, allowing cancer to be considered a controllable form of chronic disease. This treatment strategy aims to prolong patient survival, reduce pain, enhance overall quality of life, and lower associated costs.

Lung cancer continues to pose a significant risk to worldwide public health, as it is associated with high rates of mortality among diagnosed patients. For certain patient subsets, the development of next-generation targeted therapies and immune checkpoint inhibitors has resulted in long-lasting survival benefits. These treatments aim to overcome the tumor-mediated inhibition of immune cell activity, allowing immune cells to reactivate and eliminate target tumor cells. Currently, the main types of immune checkpoint inhibitors used in clinical settings are antibodies that act as inhibitors of CTLA-4 or PD-1/PD-L1. Although immune checkpoint blockage can be beneficial in treating chemotherapy-refractory malignancies and is generally well-tolerated by patients, it can also lead to immunotherapy-associated adverse reactions (irAEs) that mainly affect immune-related organs such as the liver, thyroid, skin, and intestines. Immunotherapeutic strategies represent a promising avenue to enhance the outcomes of lung cancer patients. These strategies enable the diagnosis of patients, allowing for the cure of those with early-stage disease and the conversion of advanced or metastatic lung cancer into a more manageable chronic condition (Schabath and Cote, 2019).

Due to the significant financial and societal burdens associated with diagnosing lung cancer, as well as the limited ability of current diagnostic tools to detect the disease in its early stages, the majority of lung cancer cases are diagnosed at an advanced stage. This has led to increasing efforts over the past 4 decades to enhance early detection methods for lung cancer to improve the chances of successful treatment. However, early diagnostic strategies remain unsatisfactorily and inaccurate, posing a significant obstacle to accurate diagnosis (Nooreldeen and Bach, 2021). Despite the ineffectiveness of chest radiography and sputum-based screening in reducing mortality rates associated with lung cancer, numerous studies have demonstrated that low-dose CT scans can detect early-stage lung tumors more precisely. This enables timely standardized interventions, which have the potential to significantly enhance patient survival and cure rates (Lee and Kazerooni, 2022). CT scans are considered a promising method for early diagnosis and prevention of death from malignancies. However, CT images contain a significant amount of information related to nodules, and an increase in the number of nodules makes it challenging for radiologists to accurately analyze and classify them (Thakur et al., 2020).

Positron emission tomography (PET) is incapable of providing an accurate evaluation of pulmonary nodules less than 1 cm in size or those that exhibit ground glass images on CT. False positive rates ranging from 20% to 25% have been documented when PET was utilized for the diagnosis of lung cancer. The primary cause of this is the increased intake of FDG by inflammatory cells. Furthermore, both pulmonary embolism and iatrogenic microembolism can result in FDG uptake similar to malignancy. Iatrogenic surgical procedures, such as talc pleuropexy, radiation pneumonia, esophagitis, chest tube insertion, percutaneous puncture biopsy, and mediastinoscopy, have been linked to false positive results in cases of lung cancer (Nomori et al., 2004; Chiu et al., 2011; Choi et al., 2013; Harders et al., 2014). Immuno-PET is a hybrid imaging technique that combines the fields of immunology and PET. This technique involves the use of specific antibodies or similar molecules as imaging agents to detect and identify specific cells or biomarkers of interest. Immuno-PET technique enables researchers to examine the infiltration of immune cells and understand their function in the tumor microenvironment without the need for invasive procedures. Furthermore, the application of artificial intelligence (AI) in the field of nuclear medicine has achieved significant advancements. The PET AI image processing platform uses deep learning algorithms to reduce noise, hence improving image quality, enhancing medical imaging, and improving patient experience and ease of use. Deep learning can enhance the accuracy and efficiency of tumor diagnosis by using techniques such as tissue and organ segmentation on whole-body CT images. This can also strengthen the foundation of accurate quantitative analysis and contribute to the development and implementation of nuclear medicine molecular imaging. Furthermore, the application of AI in nuclear medicine includes improvements in the quality of PET images, decreased dosage of radiopharmaceutical injections, accelerated scanning rates, and accurate detection of lesions. These improvements significantly contribute to the overall quality and efficiency of nuclear medicine imaging. Despite the limited use of AI and Raman spectral analysis technology in image processing, diagnostic assistance, and clinical applications, the future trend of interdisciplinary integration will enhance the development of optical systems, elevate the standard of medical image diagnosis, and offer patients more accurate and personalized medical services. AI can enhance the processing and analysis of extensive spectral data, resulting in improved efficiency and accuracy in data processing. AI uses machine learning algorithms to detect patterns and characteristics in the spectrum, enabling rapid classification and recognition of samples. Furthermore, AI can enhance the optimization of both the design and performance of spectral instruments, ultimately enhancing the accuracy and sensitivity of spectral analysis. The combination of Raman spectroscopy with machine learning offers the potential to develop an accurate, inexpensive, fast, and non-invasive universal medical diagnosis method, supporting both drug development and medical diagnosis (Gayap and Akhloufi, 2024).

## Applying Raman spectroscopy to screen and diagnose early-stage lung tumors

Raman spectroscopy is an analytical technique that uses the interactions between light and matter to identify the vibrational patterns of matter, which can be used to determine its unique molecular fingerprint. This method is non-invasive, does not require labeling, and is specific to polymers. As a result, it is extensively utilized for *in situ* material characterization (Fernandez-Galiana et al., 2023). Because of the distinct vibrational properties of all molecules, Raman spectroscopy holds considerable promise for use in the biochemical assessment of specific compounds of interest. Thus, Raman spectroscopy may be used to accurately analyze macromolecules, including lipids, proteins, and nucleic acids (Beton-Mysur and Brozek-Pluska, 2022). Three fundamental processes are thought to be balanced in the derivation of Einstein’s well-known Planck radiation formula: (1) the spontaneous emission of atoms, (2) the absorption of energy by atoms is proportional to the energy density in the field, and (3) the induced emission of energy by atoms is also proportional to the energy density. The third process can be described as the negative absorption of radiation. Raman, C. V. discovered that when a liquid (such as benzene) is irradiated with monochromatic light, the radiation scattered by the molecule has numerous spectral lines that change the frequency. It has been discovered that the discrepancy between the frequency of the incident and scattered light is exactly equivalent to the infrared frequency of the molecule. Therefore, the process of correcting scattering requires the absorption of radiation by the molecule. Due to numerous characteristic infrared frequencies, the molecule exhibits a comparable number of modified scattering lines. However, the spectrogram provides evidence that the liquid contains molecules with higher energy levels than usual and that when exposed to radiation, these molecules transition to a lower energy state. This phenomenon can be described as a negative absorption of radiation. (Raman and Krishnan, 1928). The Raman scattering theory is the classical theory that explains the Raman spectrum. When a photon of incident light from a monochromatic beam interacts with a molecule, it can result in either an elastic or an inelastic collision. During an elastic collision, there is no exchange of energy between the photon and the molecule. The photon simply changes the direction of motion without affecting its frequency, so that v = vo. The phenomenon of scattering in this context is referred to as Rayleigh scattering. Raman scattering is a process in which energy is exchanged between a photon and a molecule during an inelastic collision. In this process, the photon not only alters its direction of motion but also transfers some of its energy to the molecule. Alternatively, the vibration and rotation energy of the molecule can be transferred to the photon, resulting in a change in the frequency of the photon. Stokes scattering and anti-Stokes scattering are the two types of Raman scattering; v < vo represents Stokes scattering, whereas v > vo represents anti-Stokes scattering. The conventional Raman test for light primarily identifies Stokes scattering, and the disparity in frequency between the Raman scattered light and Rayleigh light is referred to as the Raman shift. The Raman shift refers to the frequency of molecule vibration or rotation, which is determined by the molecular structure and is independent of the frequency of the incoming ray. Every substance possesses unique characteristics. The Raman spectrum is directly correlated to the vibration and rotational energy levels of the molecule in a substance. It provides information about the number of Raman lines, the amount of displacement value, and the intensity of the band (Schuster et al., 2000; Enami et al., 2007; Johannessen et al., 2007).

Surface-enhanced Raman Spectroscopy (SERS) is an analytical method that combines Raman scattering techniques and Surface enhancement effects to provide exceptional sensitivity and selectivity. To achieve structural fingerprint identification of low-concentration analytes, SERS can improve the Raman scattering signal of species adsorbed on metal surfaces by several orders of magnitude compared to traditional Raman spectroscopy. SERS technology is very appealing in the field of chemical and biochemical analysis for the identification of trace compounds, but one of its key drawbacks is the low reproducibility of the signal, which limits its use as a conventional analytical approach. To improve the reliability and application range of SERS technology, researchers are constantly exploring various methods and concepts to produce strong, reproducible, SERS active surfaces. SERS technology can also be used in combination with other detection technologies, such as infrared spectroscopy, to improve the sensitivity and accuracy of surface detection. SERS technology is extensively utilized in surface and interface chemistry, catalysis, nanotechnology, biology, biomedicine, and various other fields due to its continuous enhancement and innovative features (Table 1). SERS employs the local surface plasmon resonance (LSPR) effect of a metal surface to achieve energy conversion and spectral recognition of specific biomolecules, allowing detection at the single-molecule level. SERS technology can attain exceptional sensitivity, specificity, and the ability to detect individual molecules. It can be combined with histomorphology, immunomarker analysis, and molecular hybridization to enable quantitative diagnosis at the morphological, molecular, and genetic levels. SERS technology can sensitively detect extremely subtle molecular changes in the microenvironment around early tumor cells, improving the accuracy of cancer prediction and diagnosis. SERS technology is capable of isolating and analyzing exosomes or circulating tumor DNA in the blood or body fluids of patients. Sequencing and analysis can yield significant insights into the genome of a tumor, thereby facilitating early detection of tumors, assessment of treatment efficacy, and provision of guidance for treatment selection. SERS is capable of effectively delineating the boundaries of tumors and resolving the difficulties associated with intraoperative diagnosis and residual tumor removal. Moreover, through the incorporation of nanoparticles for targeted drug release and delivery to cancer cells, SERS offers a novel strategy for drug development. SERS technology holds significant promise in the field of biomedical applications. Through continuous improvement and optimization of the SERS active matrix, probe design, and signal processing methods, the performance and application range of SERS technology can be further enhanced, providing a more accurate and efficient analytical means for cancer research and clinical environment (Li et al., 2024).

**TABLE 1:** Representative publications related to Surface Enhanced Raman Spectroscopy (SERS) and Raman spectroscopy from 2014 to 2024.

Year	Journal	Title	First author
2014	Analyst	Evaluation of Raman spectroscopy for diagnosing EGFR mutation status in lung adenocarcinoma	Wang, L
	ChemCommun (Camb)	Surface-enhanced Raman spectroscopy for simultaneous sensitive detection of multiple microRNAs in lung cancer cells	Ye, L. P
	Analyst	Quantification of an exogenous cancer biomarker in urinalysis by Raman spectroscopy	Cao, G
	ACS Appl ater Interfaces	Facile and label-free detection of lung cancer biomarker in urine by magnetically assisted surface-enhanced Raman scattering	Yang, T
2015	J iophotonics	Real-time *in vivo* cancer diagnosis using Raman spectroscopy	Wang, W
	ACS Appl Mater Interfaces	Sea-urchin-like Au nanocluster with surface-enhanced raman scattering in detecting epidermal growth factor receptor (EGFR) mutation status of malignant pleural effusion	Wang, L
	Appl Spectrosc	A Raman spectroscopic study of cell response to clinical doses of ionizing radiation	Harder, S. J
2016	Sci Rep	Raman spectroscopy identifies radiation response in human non-small cell lung cancer xenografts	Harder, S. J
	J Mater Chem B	Combination assay of lung cancer associated serum markers using surface-enhanced Raman spectroscopy	Song, C
2017	J Biophotonics	Real-time endoscopic Raman spectroscopy for *in vivo* early lung cancer detection	McGregor, H. C
	J Biophotonics	Differentiating responses of lung cancer cell lines to Doxorubicin exposure: *in vitro* Raman micro spectroscopy, oxidative stress and bcl-2 protein expression	Farhane, Z
	Anal Bioanal Chem	Monitoring doxorubicin cellular uptake and trafficking using *in vitro* Raman microspectroscopy: short and long time exposure effects on lung cancer cell lines	Farhane, Z
	Guang Pu Xue Yu Guang Pu Fen Xi	Investigations on NIR-SERS Spectra of Oxyhemoglobin for Lung Cancer Based on NIR-SERS Substrate]	Gao, F
	Sci Rep	Detection of EGFR mutation in plasma using multiplex allele-specific PCR (MAS-PCR) and surface enhanced Raman spectroscopy	Li, X
	Anal Chem	Ultrasensitive Surface-Enhanced Raman Scattering Sensor of Gaseous Aldehydes as Biomarkers of Lung Cancer on Dendritic Ag Nanocrystals	Zhang, Z
	Anal Chem	Exosome Classification by Pattern Analysis of Surface-Enhanced Raman Spectroscopy Data for Lung Cancer Diagnosis	Park, J
2018	Zhongguo Fei Ai Za Zhi	[Research Progress of Raman Spectroscopy in the Diagnosis of Early Lung Cancer]	Tian, X
	Spectrochim Acta A Mol Biomol Spectrosc	Screening and staging for non-small cell lung cancer by serum laser Raman spectroscopy	Wang, H
	J Biophotonics	Development and *in vivo* test of a miniature Raman probe for early cancer detection in the peripheral lung	McGregor, H. C
	Adv Mater	Selective Surface Enhanced Raman Scattering for Quantitative Detection of Lung Cancer Biomarkers in Superparticle@MOF Structure	Qiao, X
	ACS Sens	Correlation between Cancerous Exosomes and Protein Markers Based on Surface-Enhanced Raman Spectroscopy (SERS) and Principal Component Analysis (PCA)	Shin, H
	ACS Appl Mater Interfaces	Enzyme-Driven Switchable Fluorescence-SERS Diagnostic Nanococktail for the Multiplex Detection of Lung Cancer Biomarkers	Saranya, G
2019	Cancers (Basel)	Liquid Biopsies in Lung Cancer: Four Emerging Technologies and Potential Clinical Applications	Chudasama, D
	Spectrochim Acta A Mol Biomol Spectrosc	Raman spectroscopic discrimination of normal and cancerous lung tissues	Sinica, A
	BMC Cancer	Raman spectroscopy detects metabolic signatures of radiation response and hypoxic fluctuations in non-small cell lung cancer	•Van Nest, S. J
	J Med Imaging (Bellingham)	*Ex vivo* Raman spectroscopy mapping of lung tissue: label-free molecular characterization of nontumorous and cancerous tissues	Bourbousson, M
	Int J Nanomedicine	SERS-based differential diagnosis between multiple solid malignancies: breast, colorectal, lung, ovarian and oral cancer	Moisoiu, V
2020	Theranostics	Polymerase chain reaction - surface-enhanced Raman spectroscopy (PCR-SERS) method for gene methylation level detection in plasma	Li, X
	J Pharm Biomed Anal	Highly-selective detection of EGFR mutation gene in lung cancer based on surface enhanced Raman spectroscopy and asymmetric PCR	Guo, T
	Biomed Opt Express	Raman spectroscopy combined with multivariate analysis to study the biochemical mechanism of lung cancer microwave ablation	Song, D
	Analyst	Raman spectroscopy as a potential diagnostic tool to analyse biochemical alterations in lung cancer	Zheng, Q
	ACS Nano	Early-Stage Lung Cancer Diagnosis by Deep Learning-Based Spectroscopic Analysis of Circulating Exosomes	Shin, H
2021	J Mol Recognit	Investigations of Dianhydro-D-glucitol adsorbed on AuNPs surface: In silico and *in vitro* approach based on anticancer activity studies against A549 lung cancer cell lines	V, S
	Cancers (Basel)	Raman Spectral Signatures of Serum-Derived Extracellular Vesicle-Enriched Isolates May Support the Diagnosis of CNS Tumors	Bukva, M
	Spectrochim Acta A Mol Biomol Spectrosc	Label-free surface-enhanced Raman spectroscopy for diagnosis and analysis of serum samples with different types lung cancer	Lei, J
	Nat Protoc	Raman spectral cytopathology for cancer diagnostic applications	Traynor, D
	Nanoscale	Ultrasensitive SERS detection of exhaled biomarkers of lung cancer using a multifunctional solid phase extraction membrane	Huang, Y
	Anal Chim Acta	Ultra-sensitive and high efficiency detection of multiple non-small cell lung cancer-related miRNAs on a single test line in catalytic hairpin assembly-based SERS-LFA strip	Mao, Y
	Transl Cancer Res	The accuracy of Raman spectroscopy in the diagnosis of lung cancer: a systematic review and meta-analysis	Chen, C
2022	Lasers Med Sci	The efficacy of Raman spectroscopy in lung cancer diagnosis: the first diagnostic meta-analysis	Ke, Z. Y
	Spectrochim Acta A Mol Biomol Spectrosc	Highly accurate diagnosis of lung adenocarcinoma and squamous cell carcinoma tissues by deep learning	Qi, Y
	ACS Appl Mater Interfaces	Nanoplatform to Investigate Tumor-Initiating Cancer Stem Cells: Breaking the Diagnostic Barrier	Dharmalingam, P
	Front Chem	Diagnosis of Lung Cancer by FTIR Spectroscopy Combined With Raman Spectroscopy Based on Data Fusion and Wavelet Transform	Yang, X
2022	Biosens Bioelectron	A dual-signal amplification strategy based on pump-free SERS microfluidic chip for rapid and ultrasensitive detection of non-small cell lung cancer-related circulating tumour DNA in mice serum	Cao, X
	Nanoscale	Early detection of lung cancer via biointerference-free, target microRNA-triggered core-satellite nanocomposites	Chen, C
	Biomedicines	Lung Cancer: Spectral and Numerical Differentiation among Benign and Malignant Pleural Effusions Based on the Surface-Enhanced Raman Spectroscopy	Kowalska, A. A
	Spectrochim Acta A Mol Biomol Spectrosc	Fabrication of optoplasmonic particles through electroless deposition and the application in SERS-based screening of nodule-involved lung cancer	Wang, Z
	Biomed Res Int	Application of Surface-Enhanced Raman Spectroscopy in the Screening of Pulmonary Adenocarcinoma Nodules	Peng, B
	Analyst	Serum fingerprinting by slippery liquid-infused porous SERS for non-invasive lung cancer detection	Cai, C
	Sci Rep	Hyperglycemia and cancer in human lung carcinoma by means of Raman spectroscopy and imaging	Kopeć, M
	Biosensors (Basel)	Machine Learning Assisted Real-Time Label-Free SERS Diagnoses of Malignant Pleural Effusion due to Lung Cancer	Perumal, J
	Anal Chim Acta	Serum-based surface-enhanced Raman spectroscopy combined with PCA-RCKNCN for rapid and accurate identification of lung cancer	Cao, D
2023	Spectrochim Acta A Mol Biomol Spectrosc	Diagnoses in multiple types of cancer based on serum Raman spectroscopy combined with a convolutional neural network: Gastric cancer, colon cancer, rectal cancer, lung cancer	Du, Y
	Small	Promising Mass-Productive 4-Inch Commercial SERS Sensor with Particle in Micro-Nano Porous Ag/Si/Ag Structure Using in Auxiliary Diagnosis of Early Lung Cancer	Gao, Y
	Sens Diagn	Analyzing bronchoalveolar fluid derived small extracellular vesicles using single-vesicle SERS for non-small cell lung cancer detection	Jonak, S. T
	Nat Commun	Single test-based diagnosis of multiple cancer types using Exosome-SERS-AI for early stage cancers	Shin, H
	Nanoscale Horiz	Plasma extracellular vesicle phenotyping for the differentiation of early-stage lung cancer and benign lung diseases	Yuan, L
	J Biomed Opt	Subsecond lung cancer detection within a heterogeneous background of normal and benign tissue using single-point Raman spectroscopy	Leblond, F
	iScience	Serum laser Raman spectroscopy as a potential diagnostic tool to discriminate the benignancy or malignancy of pulmonary nodules	Luo, H
	Int J Nanomedicine	Combined SERS Microfluidic Chip with Gold Nanocone Array for Effective Early Lung Cancer Prognosis in Mice Model	Qian, Y
	Chem Phys Lipids	Raman imaging and chemometric methods in human normal bronchial and cancer lung cells: Raman biomarkers of lipid reprogramming	Kopec, M
	Biosens Bioelectron	Machine learning-assisted global DNA methylation fingerprint analysis for differentiating early-stage lung cancer from benign lung diseases	Lu, D
2024	Lu, D	Machine learning-based exosome profiling of multi-receptor SERS sensors for differentiating adenocarcinoma *in situ* from early-stage invasive adenocarcinoma	Colloids Surf B Biointerfaces
	Chem Phys Lipids	The role of glucose and fructose on lipid droplet metabolism in human normal bronchial and cancer lung cells by Raman spectroscopy	Kopec, M
	Biosens Bioelectron	3D plasmonic hexaplex paper sensor for label-free human saliva sensing and machine learning-assisted early-stage lung cancer screening	Linh, V. T. N

Raman spectroscopy produces complex and large data that usually requires multivariate data analysis. Principal component analysis (PCA), orthogonal partial least squares discriminant analysis (OPLS-DA), multivariate curve resolution (MCR), partial least squares regression (PLS), and classical least squares (CLS) are common multivariate methods. Orthogonal signal correction (OSC) and PLS can be combined and corrected using OPLS-DA. OSC can reduce the heterogeneity of clinical samples and eliminate the influence of environmental and dietary factors, both of which are crucial for the clinical application of metabonomics technology. OPLS eliminates the orthogonal variables in a given data set X and differentiates them from non-orthogonal variables, allowing for separate analysis. The OPLS approach utilizes the data from the response variable y to divide the variable x into three distinct parts in the following manner:

$$X=T_{P}P_{P}^{T}+T_{O}P_{O}^{T}+E$$ (1)

Where *T*_*P*_ represents the predicted score matrix of *X*, *P* ^*T*^_*P*_ represents the predicted load matrix of *X*, *T*_*P*_ *P* ^*T*^_*P*_ represents the predicted part, *T*_*O*_ represents the score matrix of the orthogonal component of *X* and *Y* (called OPLS component), *P* ^*T*^_*O*_ represents the corresponding load matrix, *T*_*O*_ *P* ^*T*^_*O*_ represents the part orthogonal to *Y*, and *E* is the residual matrix.

The OPLS method is implemented in two steps:

The first step is to eliminate the variables orthogonal to *Y* from the *X* data matrix, i.e.,

$$X_{P}=X-T_{O}P_{O}^{T}$$ (2)

Where *T*_*O*_ is the score matrix of the component orthogonal to Y, and *P* ^*T*^_*O*_ is the load matrix corresponding to it.

The second step is a partial least squares analysis of *X*_*P*_.

When OPLS-DA is used in Raman spectrum analysis, it can remove variation in the data that is independent of classification variables, concentrate classification information in a single principal component, make the identification model easier to explain, and facilitate the screening of Raman characteristic peaks as biomarkers.

Surface-enhanced Raman Scattering (SERS) nanoparticles (NPs) are versatile probes that can be used for tissue staining and *in vivo* imaging. They offer high sensitivity and produce unique Raman molecular fingerprints that are different from other analytical methods in different conditions (Shaffer and Gambhir, 2021). Raman spectroscopy has received significant interest as a valuable biomedical tool due to its ability to provide molecule-specific insights into changes that may be associated with the pathogenesis of cancers or other disease states (Fales et al., 2021). Researchers have successfully implemented this technology to improve the accuracy of cancer diagnosis without causing any damage to the samples or requiring the use of labels. Raman spectroscopy, instead of studying light absorption, examines the inelastic scattering of light to provide both quantitative and qualitative information about the characteristics of the substances being studied. Raman shifts occur due to changes in the energy levels of molecular vibrations caused by differences in vibration modes between ground states or chemical bonds. Raman spectroscopy can provide in-depth information about chemical structures and molecular interactions in both tumor and noncancerous tissues (D'Acunto et al., 2020).

**FIGURE 1:**
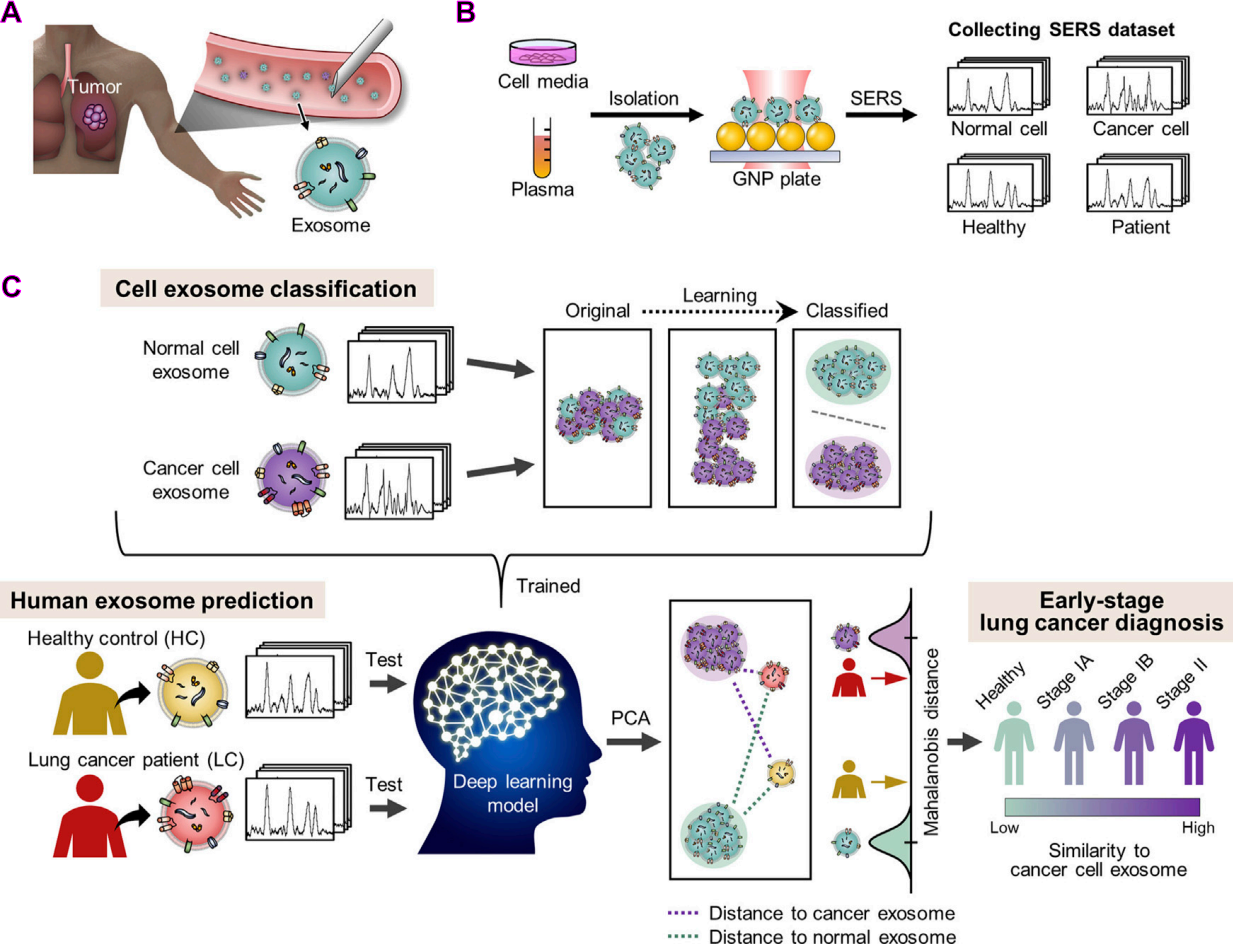
The diagnosis of lung cancer through learning-based cyclic exosome analyses. **(A)** Circulating exosomes from lung cancer patients are present in the blood. **(B)** SERS was used to collect exosome spectral data. **(C)** The basic deep learning-based approach to exosome classification and lung cancer diagnosis based upon exosome SERS signal patterns (Shin et al., 2020).

Raman spectroscopy techniques necessitate minimal intervention for the monitoring of biological tissues due to their non-destructive characteristics. Fourier transform Raman (lambda-ex 1,064 nm) and near-infrared Vision-Raman (lambda-ex 785 nm) spectroscopic analyses were used to examine *in vitro* samples of healthy and cancerous lung tissue. The researchers successfully differentiated between various sample types by analyzing their spectroscopic ratios and used relevant statistical approaches such as LDA and PCA. This study highlights the potential of combining multivariate statistics and Raman spectroscopy to assist in the diagnosis of lung cancer lesions (Sinica et al., 2019). Stimulated Raman scattering (SRS) microscopy is an analytical technique that can produce molecular maps, offering valuable insights into pathological changes in the underlying tissue. Compared to spontaneous Raman techniques, SRS provides a limited spectrum range and faster imaging rates. It focuses on capturing spectral characteristics in densely informative fingerprint regions, enabling rapid and reliable imaging. Hakert et al. developed a Time-coded (TICO) SRS microscope to assess head and neck biopsy samples using molecular contrast. This was achieved by combining a Fourier domain mode-locked (FDML) laser with a master oscillator power amplifier (MOPA), which enabled the observation of Raman transitions between 1,500 and 1800 cm^-1^. The use of electronic programming in these fiber-based lasers contributes to a strong and reliable TICO system. This system showed a high level of agreement with results from spontaneous Raman microspectrometry, providing the basis for its possible clinical application in the future (Hakert et al., 2021). SERS has been employed to distinguish between exosomes originating from various cell types (Figure 1), due to the unique composition of exosomes obtained from tumor cells in comparison to those released by healthy cells. Figure 1 demonstrates the process of extracting peripheral blood and using SERS technology to apply exosomes to each point array. After thorough drying, the signals were scanned to get representative spectra of separated exosomes in each group. Different spectral peaks in the blood of tumor patients and healthy patients could be analyzed to obtain different results. The reason for this is that SERS has been employed to identify and differentiate exosomes from various cell types. Cancer exosomes are differentiated from those formed by healthy cells by their cancer-specific molecular composition. Exosomes are formed through the process of membrane fusion and contain distinct proteins and nucleic acids derived from the parent cell. An in-depth investigation into the anchoring proteins of exosomes that are linked to the occurrence and progression of cancer, as well as the identification of specific exosomes from cancer cells, can greatly assist in the diagnosis and monitoring of cancer. SERS tags have been discovered to be capable of characterizing the GPC1, a membrane-surface anchoring protein for exosomes, particularly by analyzing its distinct characteristic peak intensity. Research on exosomes should be accelerated to generate concepts for the development of novel liquid biopsies for cancer detection and monitoring. Furthermore, protein and nucleic acid-rich exosomes from tumor cells can provide a novel method for the early detection of cancer by reflecting the dynamic changes of the primary tumor in real-time. The advancement of SERS detection approaches that are appropriate for analyzing exosome internal nucleic acids and surface proteins, achieving reproducible and high-quality Raman signals, and extracting complete diagnostic information represents a novel avenue for future studies on exosomes using SERs. SERS has been applied in numerous ways to liquid biopsies and cancer diagnostics. Several research groups have attempted to identify cancerous biomolecules in urine, blood, breath, and saliva using SERS for medical diagnostic applications. Recent studies have focused more on the SERS characterization of cancerous exosomes due to the growing importance of exosomes as biomarkers.

The regulation of specific miRNA and protease expression within tumor cells can be achieved by the tissue inhibitor of metalloproteinase-1 (TIMP-1) protein. Furthermore, the TIMP-1 levels on exosomes may serve as a viable method for the diagnosis of certain cancers. However, label-free methods for collecting reliable data about plasma exosome TIMP-1 levels are still limited (Shin et al., 2020). Lin et al. employed the synthetic CP05 polypeptide to modify a porous plasma SERS chip that could capture and differentiate exosomes from various origins. Due to its ability to precisely detect the TIMP-1 protein-associated plasmon, this SERS chip was highly suitable for analysis of the particular fingerprint peaks of exosomes. The developed SERS chip successfully differentiated between control exosomes and exosomes produced from lung and colon tumor cells. This differentiation was achieved via a combination of Raman spectroscopy and machine learning techniques, and it was performed at the single exosome level. This device serves as a practical SERS chip that can effectively apply Raman technologies to monitor and estimate the prognosis of cancer patients. Additionally, it offers a comprehensive reference for analyzing exosomal properties at the spectral level (Lin et al., 2022). Qi, Y et al. conducted a review that concluded that Raman spectroscopy is highly accurate in detecting biological tissues *in vitro* and also provides reliable data and a technical basis for *in vivo* biological research. Portable Raman systems and Raman probes can be used to acquire Raman data *in vivo*. Currently, Raman spectroscopy is mostly utilized in clinical *in vivo* detection through two methods: the combination of a Raman system with a medical endoscope for *in situ* detection, and the direct detection of living tissue using a Raman system. In general, contact devices require a smaller number of inspection sites compared to endoscopes. The contact Raman system’s exceptional adaptability enables precise contour estimation of the lesion site, thereby presenting favorable conditions for the assessment of treatment efficacy and the optimization of surgical procedures. The combination of machine learning with Raman spectroscopy is a developing field of research in the health sciences. Complex information can be extracted from spectral statistics using machine learning techniques. Machine learning models can be applied to identify the characteristics of Raman spectra and classify substances, thereby enhancing the diagnostic capability of diseases (Qi et al., 2024). Gayap, H. T. et al. conducted a review of recent studies on the use of deep learning methods for lung cancer detection and diagnosis. In every investigation, the sensitivity, accuracy, and specificity of lung cancer diagnosis in CT scans were consistently higher with deep learning models than with typical machine learning techniques. Traditional cancer diagnostic approaches, such as visual inspections and biopsies, are often time-consuming, subjective, and prone to errors. In contrast, deep machine learning (ML) can detect cancer with greater accuracy than conventional methods. Deep machine learning algorithms are trained on extensive datasets of medical text, images, and other data, enabling them to acquire the ability to identify patterns that are invisible to humans. Deep learning algorithms provide a distinctive capability to automatically acquire complex patterns and characteristics from complex data sets, facilitating the development of accurate and robust cancer detection models. These algorithms, which have been trained on extensive medical image datasets, can identify small differences that signify the progression of cancer, thereby enabling early detection and intervention (Gayap and Akhloufi, 2024). Bioactive gold nanomaterials exhibit considerable promise in the identification and visualization of disease biomarkers, particularly in the development of diverse biosensor designs, electrochemical analysis sensors, and color rendering analyses. Gold nanoparticles have a significant role in the treatment of lung cancer, particularly in photothermal and photodynamic therapy. This is attributed to their inert and stable nature, lack of immunogenicity, and reduced cellular toxicity. Furthermore, the increased permeability and retention effects of gold particles, their functionalized derivatives, and drugs loaded with gold particles enable transvascular penetration through tumor blood vessel walls, thereby promoting accumulation within tumor cells. Arifuzzaman, Md., et al., demonstrated the potential of gold nanoflower-labeled magnetic nanoparticles (FeO) with SERS activity for the detection of carcinoembryonic antigen (CEA). Gold nanoparticles with plasma-enhanced Raman characteristics can be used to investigate intracellular drug release profiles and visualize cancer cells (Arifuzzaman et al., 2024). Kopic et al. evaluated the biochemical properties of specific organelles within CCL-185 lung cancer cells by supplementing them with glucose and deuterated glucose. The authors were able to elucidate the distinctive biochemical landscape of these tumor cells and provide evidence for the glucose metabolism activity of these cells under normoglycemic and hyperglycemic conditions by employing isotope replacement cells. The authors concluded from these analyses that deuterated glucose effectively distinguished between exogenous dietary lipid uptake and *de novo* lipid biosynthesis when glucose was substituted with deuterated glucose. The Raman signal corresponding to cytochrome C concentrations at 1,583 cm^−1^ can be utilized to monitor mitochondrial metabolic activity. These findings provide direct support for the ability of high glucose levels associated with hyperglycemia to suppress mitochondrial metabolic activity within cancer cells via oxidative phosphorylation, implying that hyperglycemia can promote greater tumor cell malignancy by inhibiting apoptosis and oxidative phosphorylation (Kopec et al., 2022).

To obtain a device that could accommodate uneven surface topologies in human and animal tissues and imperfect centering during endoscopy, Zavaleta et al. inserted a 5 mm-diameter non-contact, flexible, fiber-based device into an existing endoscope. The operating distance of the device was adjusted between 1 and 10 mm. After obtaining approval from the Institutional Review Board (IRB), they successfully administered SERS labels topically to patients and used their devices during colonoscopies. Liu et al. employed a comparable method by locally administering a mixture of multiple SERS labels to a mouse model of esophageal cancer. They successfully visualized tumor tissue within the esophageal cancer lumen and accurately measured the expression level of the biomarker using a micro-spectral endoscope equipped with rotary scanning and axial pull-back. Currently, the primary constraint on this method is the lowest possible fiber diameter, which also restricts the endoscope, because of constraints in the development and application of excitation and detection optics. Upon resolving this issue, additional applications beyond cancer detection may be viable, including upper respiratory tract screening for viral particle detection. Liquid biopsies provide the potential to simplify and reduce the discomfort associated with early disease diagnosis and monitoring for patients. Specifically, to evaluate the probability of the existence of disease biomarkers. Fluid substitutes for tissue have the potential to continuously track the development of diseases and the effectiveness of treatment, without the need for imaging techniques that may not be capable of detecting changes in tumor size. Li et al. recently developed and executed a SERS immunoassay to differentiate between healthy individuals and patients with pancreatic tumors, as well as to detect metastatic from non-metastatic disease, by analyzing 2 μL exosomes in clinical serum. Using alterations in the orientation of Raman reporting factors upon binding to a target, Alvarez-Puebla et al. incorporated a SERS platform to monitor the biomarker c-MYC, a transcription factor that is dysregulated in 70% of cancers. The sensor was validated in healthy donors, cell lines, and one cancer patient. SERS analysis possesses non-destructive and single-molecule detection capabilities, rendering it an exceptional tool for highly sensitive biomedical applications. These applications include the identification of disease biomarkers, pathogenic microbes, circulating tumor cells (CTCs), and monitoring therapeutic drug levels. To make the marker-free SERS technique more useful, Shin et al. (2020) showed that a deep learning algorithm can discriminate between healthy and lung cancer cell lines using SERS signals from their exons with a 95% accuracy rate. Exosomes isolated from lung cancer cells and those extracted from individuals with the disease were found to be 90.7% identical according to their method. This label-free SERS technique based on deep learning and minimal invasiveness can therefore be applied to additional SERS-based biosensing applications, thereby enhancing its potential for clinical translation. In comparison to alternative analytical methods, SERS provides numerous benefits, including enhanced detection limits and multiplexing capabilities. Various AI and ML techniques have been extensively employed to enhance the thorough and accurate classification of samples by analyzing data that was previously acquired by SERS. These methods facilitate the classification of small chemicals, fungal cells, antiviral drugs, infectious disease diagnostics, protein antigens, mammalian cells, bacterial cells, and extracellular vesicles (Langer et al., 2020; Panikar et al., 2021; Mahmoud et al., 2023).

## The use of Raman spectroscopy for early lung cancer screening, diagnosis, and treatment

Lung cancer is the leading cause of cancer-related deaths, with an average 5-year survival rate of only 15% for those affected. A significant risk factor associated with lung cancer is smoking, and this disease is responsible for more deaths each year than pancreatic, breast, colorectal, and prostate cancers combined. Lung cancers are broadly categorized as either small cell or non-small cell cancers (e.g., squamous cell carcinoma, large cell carcinoma, adenocarcinoma). The specific signs and symptoms reported by individuals may differ depending on the type of tumor and the stage of metastatic progression. Diagnostic, staging, lung function testing, and screening for potential metastases are all crucial in patients with suspected lung cancer.

The precise molecular mechanisms responsible for the development and progression of lung cancer are not yet fully understood. However, non-coding RNAs (ncRNAs) play a crucial role in facilitating several tumorigenic processes. Circular RNAs (circRNAs) are now recognized as important targets in lung cancer research. These ncRNAs form covalently closed loop structures that lack a 5′cap or 3′poly-A tail by undergoing intron or exon cyclization. These distinctive characteristics make them resistant to exonuclease activity, resulting in greater stability compared to linear RNAs. circRNA expression patterns vary depending on the species, tissue, and time. These variations make circRNAs potential candidates for diagnostic and therapeutic targeting. The precise underlying mechanisms of circRNA dysregulation in lung cancer remain poorly understood, despite increasing evidence indicating its frequent occurrence and close association with the progression of the disease. Specific circRNAs are believed to impact the proliferation, apoptosis, immune escape, migration, and resistance to drugs in tumor cells by activating various signaling pathways (Wang CD et al., 2020). Lung cancer cell-derived exosomes are rich in different types of lipids, proteins, RNAs, and other macromolecules, and their uptake by recipient cells can facilitate intracellular information transfer. Given their natural status as biomolecular transporters, these lung cancer cell-derived exosomes have been demonstrated to stimulate the formation of angiogenesis within tumors and influence the tumor microenvironment (TME) in a way that supports tumor growth. The modified protein and miRNA expression patterns observed in these exosomes can be effectively targeted to diagnose lung cancers, making these membrane-enclosed vesicles attractive diagnostic, prognostic, and therapeutic targets. Exosomes play a crucial role in liquid biopsy-based strategies for lung cancer detection due to their potential to impact angiogenesis, antitumor immunity, and drug resistance. Exosomes have potential as biodegradable agents for targeted drug delivery in therapeutic applications due to their biocompatibility, non-toxicity, lack of immunogenicity, and capacity to circulate for long durations, cross biological barriers, and be internalized by recipient cells (Li et al., 2021). Yang et al. utilized data combination and wavelet transformation, combined with Fourier transform infrared (FTIR) spectroscopy and Raman spectroscopy, to examine serum samples from lung cancer patients and healthy controls. The Raman spectra of these samples provided a more profound biological understanding compared to the FTIR spectra. The spectral data underwent processing using an optimized wavelet threshold denoising (WTD) method. Subsequently, a partial least squares discriminant analysis (PLS-DA) model was applied. The results showed an accuracy of 93.41%, specificity of 96.08%, and sensitivity of 90% for the combined data in the predictive dataset after WTD processing. Therefore, these approaches are very suitable for the diagnosis of lung cancer, making them potential targets for future use in clinical screening and diagnostic applications (Yang X et al., 2022). Chen et al. performed a comprehensive review and meta-analysis to validate the high sensitivity and specificity of Raman spectroscopy as a noninvasive method for diagnosing lung cancer. Their findings support the promising clinical potential of this technology. (Chen C et al., 2021).

**FIGURE 2:**
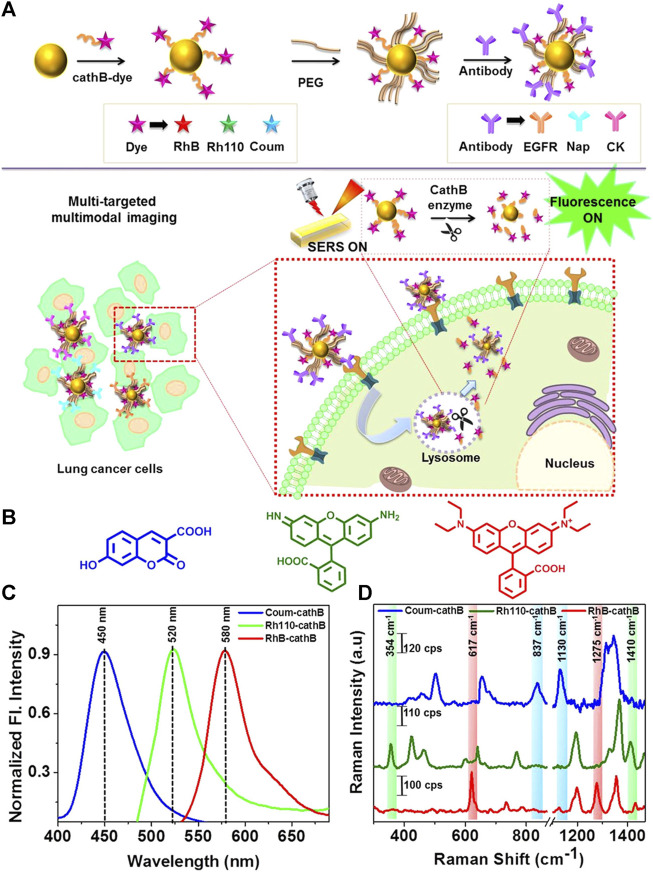
**(A)** Schematic overview of the enzyme-triggered antibody functionalization FSENP switching for the detection of multiple lung cancer-biomarkers. **(B)** The structural properties of Rhodamine 110 (green), Rhodamine B (red), and 7-hydroxy-3-carboxycoumarin (blue). **(C)** Fluorescence and **(D)** SERS analysis of 7 *µ*M Rh4-cathB, RhB-cathB, and Coum-cathB in PBS. Emission spectra were obtained using Coum-cathB, Rh110-cathB, and RhB-cathB excitation at 360, 475, and 520 nm, respectively, whereas SERS spectra were obtained using excitation at 633 nm (Saranya et al., 2018).

Microwave ablation (MWA) is a therapeutic approach that utilizes high-frequency electromagnetic waves to induce thermogenesis, necrosis, degeneration, and microvascular thrombosis within tumor cells by vibrating polar molecules, predominantly water molecules. This vibrational process subsequently causes protein coagulation and necrosis, degeneration, and microvascular thrombosis. This results in a sharp drop in intratumoral blood perfusion, causing ischemic necrosis that can inactivate tumors and modulate therapeutic effects throughout the body. Song et al. used confocal Raman microspectral imaging to investigate the effects of MWA on lung squamous cell carcinoma (LUSC). They examined the spectral properties of samples from 12 treated patients to identify alterations related to disease progression and distinguish them from changes observed in healthy lung tissues after MWA. The sections before and after the MWA were used to produce Raman datasets for site-scanning purposes. Significant biochemical changes within these sections were subsequently identified using K-means cluster analysis (KCA) and principal component analysis (PCA) methods. The post-MWA group exhibited spectral changes related to collagen, nucleic acids, tryptophan, and phenylalanine in comparison to the pre-MWA group. The results of this study indicated that MWA treatment leads to an increase in protein, lipid, and nucleic acid levels in tumor tissues. Additionally, the comparative spectral assessment provided further evidence that MWA does not have any detrimental impacts on paracancerous tissues. The results indicate that lung tumor tissues show significant biochemical variation in response to MWA treatment. This technique demonstrates the possibility of analyzing biochemical reactions during thermal ablation, which can be used to improve the efficacy of MWA in treating lung cancer (Song et al., 2020). Cathepsin B (cathB) is a protease that plays a crucial role in the invasiveness and progression of tumors. It functions by breaking down tripeptide sequences under acidic pH conditions within the TME in specific probe-based applications (Figure 2). The enzymatic cleavage, which is sensitive to pH, activates a previously inactive fluorophore by disrupting its coupling with the gold nanoparticle (AuNP) surface. This disruption occurs due to an increase in the distance between the fluorophore and the AuNP surface. As a result, the activity of SERS is slowed down, inhibiting the enhancement caused by the nano-coarse-metal matrix. Researchers used fluorescent SERS-encoded nanoparticle probes (FSENPs) to identify protein targets by modifying these probes with monoclonal antibody subunits. The resulting fingerprint SERS peaks at 837, 354, and 617cm^-1^, along with probe emission peaks at 450, 520, and 580 nm, were used to simultaneously, sensitively, and specifically identify numerous biomarkers of interest (Saranya et al., 2018). Enzyme-driven fluorescence SERS-encoded nanoparticle probes can perform sensitive and specific analysis of disease targets using a multi-mode approach, making them a very efficient system for detecting biomarkers. The interaction with the cathB enzyme enhances the potential for accurate and simultaneous identification of numerous targets. A switching mechanism is used to produce SERS and fluorescence. Multicolor image-guided spectral tracking facilitates the precise identification of individual and multiple biomarkers in complex biological environments via this effective bimodal strategy. The semi-quantitative evaluation of the biomarkers using these two methods revealed that the levels of EGFR, CK, and Nap in cancer cells were approximately 15, 8, and 1.2 times higher, respectively, compared to normal cells. Furthermore, because of the significant influence of nanotechnology in the field of cancer detection and treatment, these intricately designed nanostructures are anticipated to serve as effective platforms for high-throughput screening to assist in the early diagnosis of disease.

## Raman spectroscopy as a tool to screen, diagnose, and treat early-stage lung adenocarcinoma

Despite significant progress in the identification and management of lung cancer, it remains a leading cause of cancer-associated mortality. Lung adenocarcinoma (LUAD) is the predominant histological subtype observed in individuals with lung cancer. It is crucial to possess an in-depth understanding of the pathology, diagnosis, and treatment of these tumors to enhance patient outcomes (Hutchinson et al., 2019). CT scanning can detect the early stages of LUAD development by identifying ground glass nodules, which can eventually grow into invasive lung adenocarcinoma. The implementation of lung cancer screening strategies has led to increased testing for these lesions, which has prompted significant inquiries about the progression of the disease as well as suitable methods of surveillance and treatment. Succony et al. (2021) investigated the involvement of the immune system in the development of adenocarcinoma. They focused on pre-invasive lesions, which have clear characteristics, as a potential target for early-stage treatment of LUAD before it progresses to invasive lung cancer. Investigations into the molecular changes linked to each stage of the advancement process, particularly those that induce the mutation of the EGFR and KRAS genes, are crucial for gaining a deeper comprehension of the development of invasive adenocarcinomas. Due to the complex and diverse nature of EGFR and KRAS gene mutations, it is crucial to classify these mutations to gain a deeper understanding of the development of preinvasive diseases and to accurately anticipate the efficacy of targeted therapy.

Gaining deeper insight into interactions between tumors and the immune system is crucial for the development of new therapeutic methods (Succony et al., 2021). Immune checkpoint blockade (ICB) approaches have revolutionized the treatment of LUAD. However, there is now a limited comprehension of the immunological subtypes of LUAD and their correlation with ICB responses in clinical settings. To address this knowledge gap, Wang et al. performed consensus molecular typing using non-negative matrix factorization (NMF) and molecular subtyping and association analyses of LUAD using The Cancer Genome Atlas (TCGA) and nine validation datasets. Two LUAD subtypes with high-risk and low-risk survival outcomes were identified and ICB treatment responses were predicted using the TIDE algorithm. Validation datasets demonstrated that high-risk LUAD samples had lower TIDE scores, increased PD-L1 expression, and a higher tumor mutational burden (TMB). These samples also showed mutations in the *CDK4*, *CDK6*, and *TP53* genes. Therefore, ICB treatment could be a feasible strategy to treat individuals with the high-risk subtype of LUAD (Wang QH et al., 2020). Ground-glass opacity (GGO)-related lung cancer is a radiologically distinct form of disease that typically has a less serious course and is associated with improved survival outcomes, implying that GGO tumors have distinct biological properties. However, there have been few attempts to successfully and systematically characterize the immunological and molecular properties of GGO-associated pulmonary nodules. Chen et al. combined multi-omics techniques, such as targeted gene sequencing, RNA-Seq, T cell receptor sequencing, and the detection of circulating tumor DNA, to accomplish 3D image reconstruction to facilitate the quantitative evaluation of GGO-based molecular and immune characteristics. In general, GGO-associated lung tumors have a smaller number of mutations compared to solid nodules. Furthermore, immunofluorescent staining confirmed that GGO components exhibited reduced immunoenvironmental and immune pathway activity, as well as decreased infiltration by the majority of immune cell subpopulations, in transcriptomic analyses. Sequencing of T cell banks revealed that instances of GGO-associated lung cancer exhibited minimal T cell expansion. In comparison to non-GGO tumors, HLA heterozygosity loss was considerably lower in cases of GGO-type LUAD. During the analysis of circulating tumor DNA, it was observed that the release of tumor DNA into the peripheral circulation is directly correlated with the size of non-GGO components of the tumor (Chen KZ et al., 2021).

The ribonucleotide reductase subunit M2 (*RRM2*) has the potential to be a useful prognostic biomarker in various types of cancer. Jin et al. utilized TCGA data to investigate the association between *RRM2* gene expression and prognostic outcomes in LUAD patients by assessing the correlations between gene expression and specific clinical characteristics or survival outcomes. Ultimately, they concluded that patients with LUAD had a notable increase in *RRM2* expression compared to healthy lung tissues. Furthermore, the overexpression of *RRM2* was significantly associated with tumor stage and TNM classification. Multivariate analyses demonstrated that *RRM2* upregulation was independently associated with overall survival. Furthermore, GSEA analyses indicated that patients with high levels of *RRM2* exhibited enrichment in various biological processes and pathways, including cell cycle, p2 signaling pathway, DNA replication, small cell lung cancer, apoptosis, and cancer pathways. Overexpression of *RRM2* significantly increased the invasiveness of LUAD cells and led to elevated activity in the Bcl-2 and e-cadherin signaling pathways, while simultaneously reducing p53 signaling. High levels of *RRM2* expression can thus independently predict poor LUAD patient outcomes (Jin et al., 2020).

**FIGURE 3:**
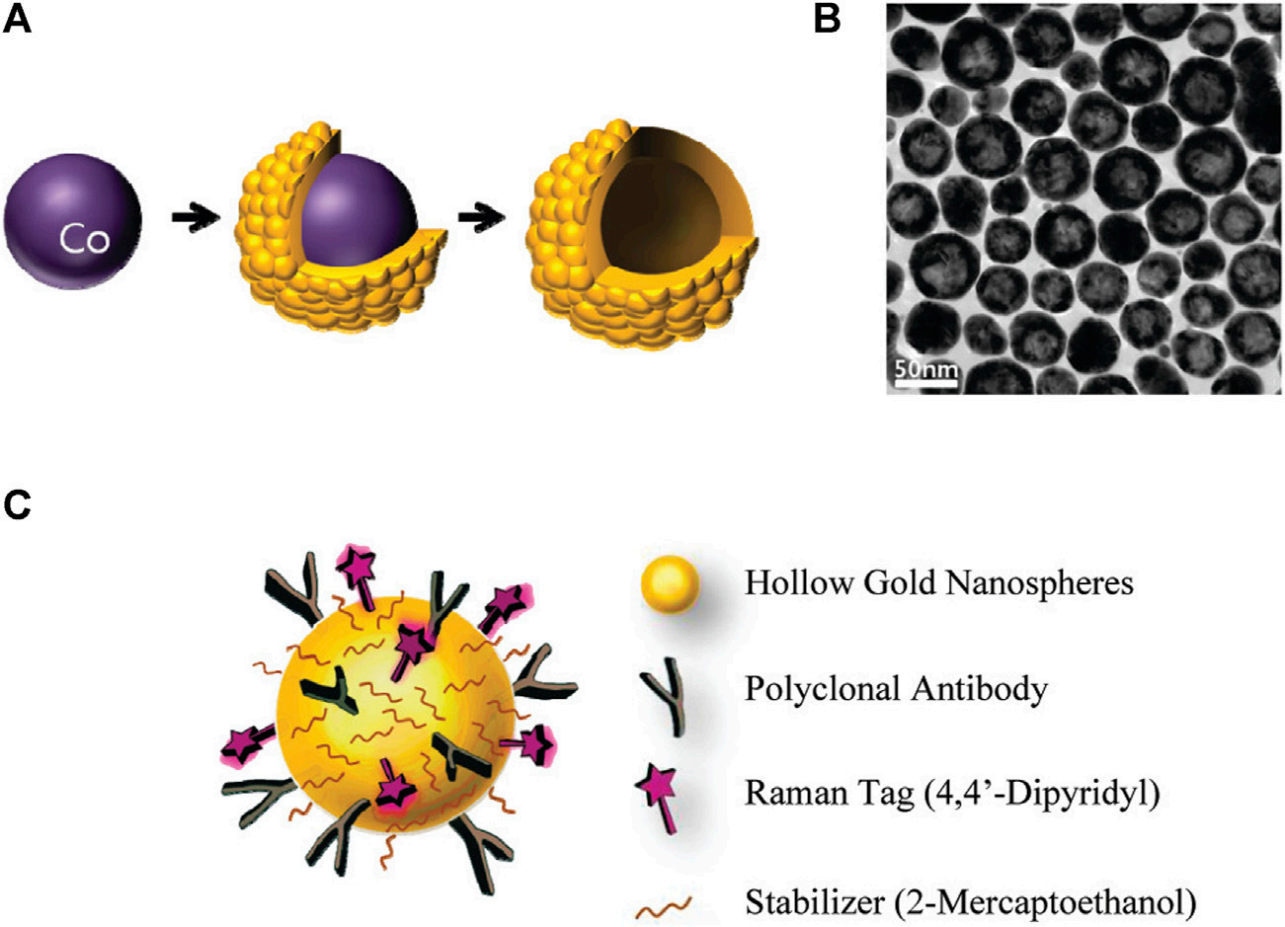
Schematic overview of the use of HGNs for SERS immunoassays. **(A)** Experimental approach to HGN generation from cobalt nanoparticle templates. **(B)** TEM images of prepared HGNs, and **(C)** Raman label (4,4′-bipyridine) and CEA antibody coupling to these HGNs (Chon et al., 2009).

SERS techniques have been used to differentiate between lung nodules and healthy patients using serum samples, with intraoperative biopsy results as a reference. Peng et al. obtained serum samples from 116 patients with lung nodules of less than 3 cm in diameter. Among these patients, 58 were diagnosed with benign nodules and 58 were diagnosed with lung tumors based on pathological examination. Samples of serum were also obtained from 63 healthy control subjects, and gold nanorods were selected as a SERS substrate. Support vector machine (SVM)-based machine learning-based sample classification was conducted, and cross-validation was performed on the resulting SVM model. The researchers observed distinct disparities in the SERS spectra when comparing the samples from tumor, benign nodule, and healthy control patients. The model ultimately achieved an accuracy of 93.33% in differentiating among these three groups of samples. The results highlight the potential of using nanoparticle-based SERS techniques in combination with SVM to assist in clinical diagnostic efforts and screening for LUAD (Peng et al., 2022). Hollow gold nanospheres (HGNs) have substantial single-particle enhancement effects, with hotspots localized on the pinholes found inside their structure (Figure 3). Due to their distinct characteristics, HGNs can be employed in reliable immunoassays specifically developed to identify cancer markers. Magnetic beads are used as a supporting substrate to facilitate the formation of immune complexes. These SERS-based immunoassay strategies can address the issue of slow immune response due to limited diffusion kinetics on solid substrates, as these reactions take place in solution. The resultant SERS-based immunoassay provides effective and reproducible results based on the application of HGNs and magnetic beads. The detection of marginal tissue is the final and most important step in the surgical resection of lung tumors (Chon et al., 2009). This class of SERS nanotags utilizes 1,280 nm excitation. It consists of the combination of chowopyran dyes that are capable of reporting molecules, together with HGNs that exhibit unprecedented properties. These nanotags exhibit a LOD within the picomolar range. Dye molecules possess distinct NIR reporter genes due to their ability to form strong bonds with the gold surface of HGN through several chow-group attachment groups, leading to a highly pronounced SERS signal. SERS nanotags based on the combination of chaeopyran dyes and tunable HGN showed unexpectedly superior performance compared to dyes using more commonly used gold nanoparticles or HGNS with traditional Raman reporter genes like BPE. This significant finding now enables the use of SERS nanotags in many optical applications, such as deep tissue characterization. It will also serve as the foundation for future research that utilizes their unique spectral properties. HGNs provide a unique combination of characteristics that make them very suitable for many biomedical uses. However, they exhibit significant sensitivity to changes in their environment and possess a propensity to accumulate, particularly when subjected to elevated salt levels or changes in pH. This characteristic is unfavorable for applications such as drug delivery and cell imaging, where the presence of stable solutions is crucial for efficient cell absorption. Therefore, it is imperative to identify appropriate stabilizers for HGNs. However, no previous comparisons have been made about prospective stabilizers for these nanostructures. This study introduces an enhanced technique for stabilizing HGN that effectively shifts the SPR from around 700 nm–800 nm or beyond. To determine the most effective stabilizer for HGN, this review compares three commonly used stabilizer materials: polymers, sugars, and silica. In addition to SEM imaging, the analysis was conducted using dynamic light scattering and extinction spectroscopy. The findings indicated that PEG had the highest efficacy as a stabilizer for HGNs, demonstrating enhanced resistance to variations in salt concentration and pH value, as well as improved long-term stability in solution. Furthermore, it has been shown that SERS detection can be accomplished at both 1,064 and 785 nm excitations, in addition to enhancing stability. The enhanced stability of the SPR in the near-infrared (NIR) region, along with the ability to detect SERS, indicates that these nanostructures have significant promise for applications in biological SERS imaging and drug delivery.

**FIGURE 4:**
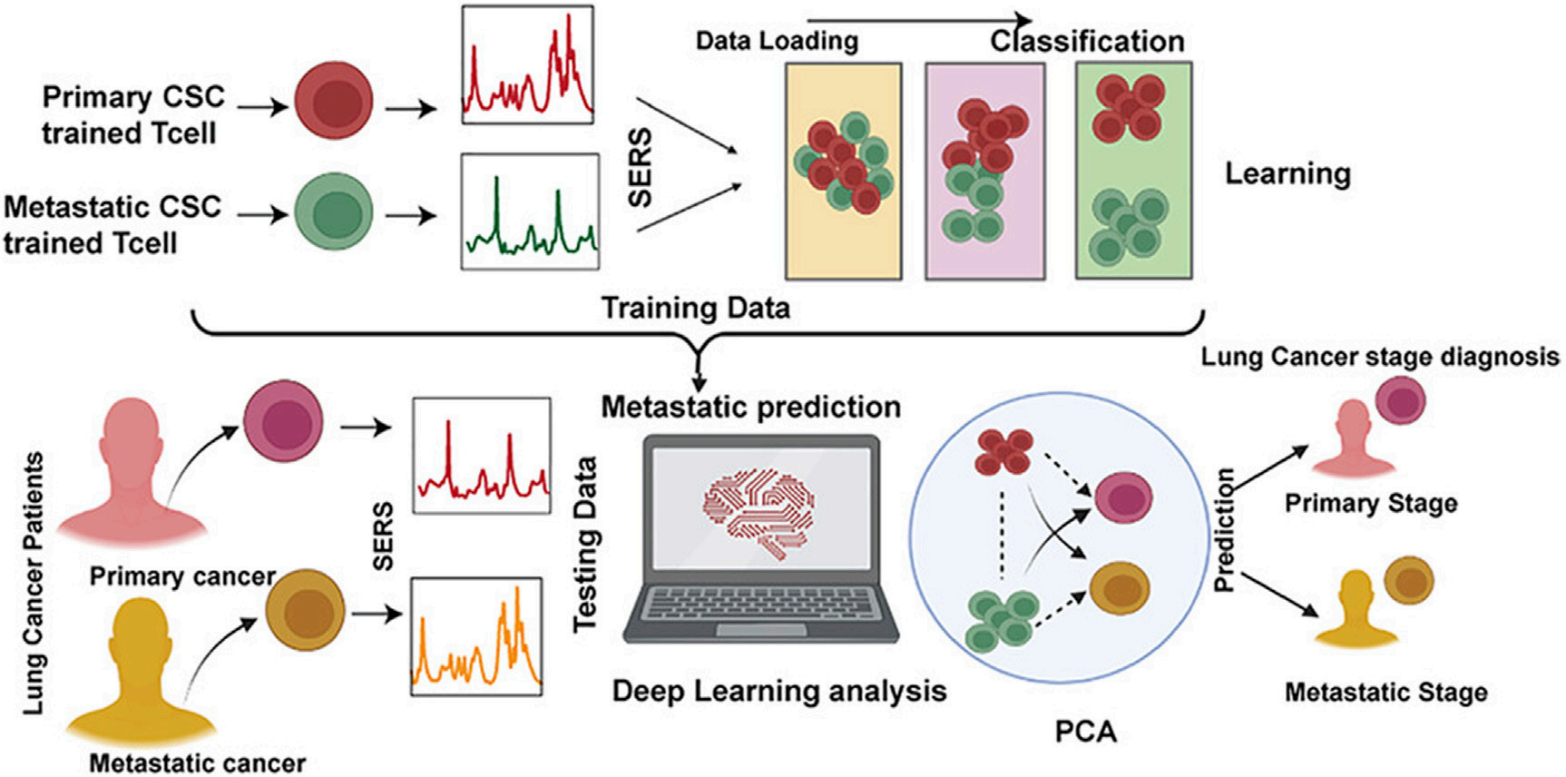
The application of ultrasensitive nanosensors for the mapping of the bidirectional interactions between tumors and the immune system to support accurate lung cancer diagnosis (Ganesh et al., 2023).

**FIGURE 5:**
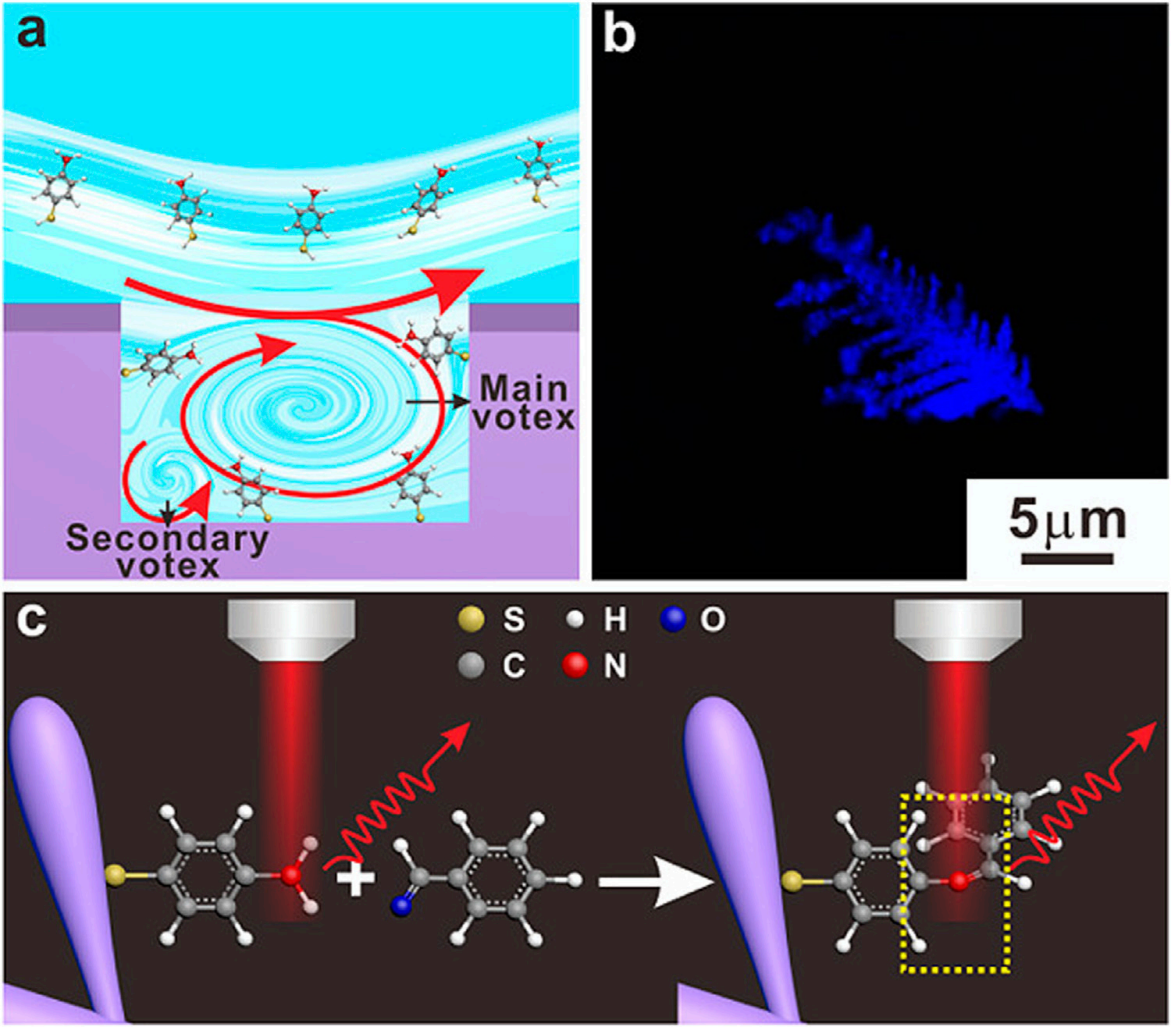
The design of an ultra-sensitive SERS sensor for gaseous aldehydes as a biomarker to detect lung cancer using dendritic silver nanocrystals (Zhang et al., 2017). **(A)** Sketches of the cavity flow dynamics. **(B)** Optical images of dendritic Ag nanocrystals. **(C)** Schematic illustration of the strategy to detect aldehydes.

Qi et al. utilized Raman spectral signals and transformed them into two Villaman spectral graphs using short-time Fourier transformation. This approach resulted in deep learning models that were highly accurate for identifying adenocarcinoma and squamous carcinoma, achieving an accuracy rate of over 96%. The models also demonstrated a sensitivity of 95% and a specificity of 98%. These models outperformed traditional PCA-LDA models for adenocarcinoma (with an accuracy of 0.896, sensitivity of 0.867, and specificity of 0.926) and squamous cell carcinoma (with an accuracy of 0.821, sensitivity of 0.776, and specificity of 1.000). Convolutional neural network (CNN)-based models have significant benefits in language processing and image recognition. They can also enhance the diagnostic classification of lung tissue samples, making it easier to diagnose LUAD and LUSC samples accurately. This, in turn, helps assess surgically resected marginal tissues with a high level of diagnostic accuracy (Qi et al., 2022). Raman spectroscopy can also be used to compare fresh lung tissue samples with histological specimens. In a particular study, researchers identified variations in the molecular composition of normal lung tissues, as well as samples of LUAD and LUSC, using Raman mapping. The K-means clustering technique successfully captured the molecular heterogeneity of the tissue samples, which was further validated by analyzing the correlations with corresponding hematoxylin and eosin (H&E)-stained tissue sections. Even in sections that appeared to have consistent H&E staining, there were noticeable differences in signal backgrounds between normal and tumor tissues. This shows that Raman spectra can distinguish between cancerous and healthy lung tissues. Thus, Raman classification models can be developed for lung tissues by analyzing the differences in Raman spectra at the microscopic level, allowing for more effective disease diagnosis and treatment stratification (Bourbousson et al., 2019). Through the use of the distinct interactions between T cells and tumor cells, it is potentially feasible to enhance the detection of lung cancer using an immunodiagnostic method (Figure 4). To that end, Ganesh et al. designed an ultra-sensitive T-sense nanosensor that employed multi-photon ionization to detect particular diagnostic features of interest. The researchers utilized lung cancer cells, CSC-associated T cells, and cancer-associated T cells obtained from patients with primary and metastatic lung cancer. They constructed *in vitro* predictive models that demonstrated clear molecular, functional, and phenotypic distinctions between patient-derived T cells and healthy samples for use in an immunodiagnostic setting. The authors adopted a machine learning model that was trained with co-cultured T cells using SERS data from preclinical models using primary and metastatic lung cancer cells. This allowed them to capture the distinct characteristics associated with patient-derived T cells, providing a more comprehensive understanding of the diversity and complexity of tumor-associated T cell features in patients. Furthermore, they demonstrated the practicality of clinical immunodiagnostic methods by applying their approach to an independent patient sample cohort. These data indicate that immunodiagnostic strategies can be applied to diagnose cancers in clinic settings and to determine effective clinical care strategies for T cell-related diseases (Ganesh et al., 2023). Using molecular and phenotypic alterations in tumor-associated T cells, Ganesh S. et al. developed an immunobiomarker for the diagnosis of primary and metastatic lung cancer. Preclinical models of primary and metastatic lung cancer (H69-AR and NCI-H1915, respectively) were utilized to identify molecular and phenotypic alterations in tumor-associated T cells. Based on SERS-trained machine learning models, the similarity between cancer-associated T cells (CAT) generated *in vitro* and CSC-associated T cells (CSCAT) and T cells obtained from patients diagnosed with primary and metastatic lung cancer (PDT) was assessed. The immunodiagnostic features were demonstrated to be reliable. The study data revealed that cells in peripheral blood display distinct phenotypes associated with primary and metastatic lung cancer. The prediction approach using peripheral blood T cells in patients showed a high level of accuracy in diagnosing primary lung cancer, with a specificity of 94.1% and a sensitivity of 100%. For metastatic lung cancer, the method exhibited a specificity of 97.9% and a sensitivity of 94.4%. The findings illustrate that nanosensor-assisted immunodiagnostic characteristics possess the capability to accurately detect primary lung cancer and assess the possibility of metastasis in this tumor (Ganesh et al., 2023). Kowalska et al. combined SERS and PLS algorithms to rapidly diagnose lung cancer and distinguish between lung tumors and cases of pleural effusion. They determine that PLS approaches are the most effective for testing various configurations of SERS data. The calculated area under the ROC curve values was in agreement with the RMSE results, confirming the validity of the PLS approach and highlighting the feasibility of combining SERS methodology and PLS analyses to detect lung tumors and pleural effusion (Kowalska et al., 2022). Furthermore, Figure 5 shows another useful use of SERS-based analytical methods: it shows the finding of gaseous Raman weak aldehydes, which are thought to be biomarkers linked to elevated amounts of volatile organic compounds in lung cancer patients. To achieve enhanced gaseous molecule adsorption, researchers generated dendritic silver nanocrystals with a structure similar to moth antennae. These nanocrystals have many cavities, which allow for a longer reaction time between these gaseous molecules and the solid nanocrystal surface via the “cavity vortex” effect. The capture and detection of gaseous aldehydes at extremely low concentrations, measured in parts per billion, was accomplished using a nucleophilic addition reaction. This procedure involved the use of p-aminothiophenol (4-ATP) as a Raman active probe molecule that was pre-grafted with dendritic silver nanocrystals. This SERS-based approach for detecting biomarkers related to lung cancer is not influenced by humidity. Therefore, it is an optimal, noninvasive, fast, cost-effective, and simple method for identifying lung malignancies (Zhang et al., 2017). The limited sensitivity in identifying molecules with weak Raman signals and the low ability of gas molecules to adhere to solid substrates are the primary obstacles preventing the widespread use of SERS in gas detectors. The adsorption gas molecules on solid characteristics of the substrate can be tailored by manipulating the surface morphology of the substrate. Gas molecules demonstrate distinct adsorption characteristics on a flat substrate as opposed to a rough system due to their fast diffusion rate. Zhang, Z. et al. utilized a dendritic silver nanocrystal base to establish a reliable SERS method for quantifying typical lung cancer biomarkers of aldehydes, drawing inspiration from the structural attributes of feathered antennae of moths (dendritic). As a result of the presence of numerous hole traps that possess unique structural attributes, gaseous aldehydes traverse the dendritic surface and encounter the “hole vortex” phenomenon, which lengthens the duration of interaction between the surface and the gas. A bridge is formed between gaseous aldehydes by the Raman-active 4-ATP molecule. 4-ATP facilitates its adsorption on dendritic silver nanocrystals through mercaptan groups, hence enabling the interaction of amine groups with other functional molecules. When aldehydes reacted with the Raman active probe molecule 4-ATP through nucleophilic addition, the SERS signal of 4-ATP underwent considerable changes, allowing for detection at the part per billion (ppb) level. The capability to identify Raman weakly strong gaseous aldehydes with high sensitivity using SERS holds significant potential for early screening of lung cancer (Zhang et al., 2017).

## Raman spectroscopy as a tool to screen, diagnose, and treat early-stage lung squamous cell carcinoma

LUSC is a subtype of non-small cell carcinoma that accounts for approximately 40% of all lung cancer cases. The limited availability of targeted therapeutic options, in comparison to those utilized for lung adenocarcinomas, generally results in unfavorable outcomes for patients with this subtype. Indeed, specific biomarkers of LUSC and important targets for the precision treatment of these tumors are unknown (Li et al., 2018), with their characteristics sufficiently different from LUAD to limit similar benefits from targeted treatment. The development of immunotherapeutic regimens has resulted in better results for patients with LUSC. Additionally, the production of more comprehensive proteomic datasets has the potential to highlight new treatment targets (Niu et al., 2022). Comparisons of several forms of SCC have shown that these tumors have comparable mutational landscapes. Mutations are frequently detected in the *TP53, TP63, SOX2, CDNK2A (P16-INK4A), KMT2D, NOTCH1, PIK3CA,* and *PTEN* genes. Comparative research at the genomic level showed that LUSC and ESCC had more similarities with each other than with other SCCs. Additionally, these tumors have more shared characteristics than adenocarcinomas derived from the same tissue of origin. These analyses of SCC origins have also indicated that the original tumor cells are caused by mutations in proliferating basal cells, which are capable of self-renewing and generating terminally differentiated cells. A greater understanding of how these initial cells give rise to cancer stem cells will aid the development of innovative techniques to avoid tumor progression (Sanchez-Danes and Blanpain, 2018). Although ICB treatment has proven to be advantageous in managing LUSC, it does not effectively benefit a significant subset of patients with high levels of tumor PD-L1 expression. To better understand the mechanistic basis for therapeutic resistance, it is critical to fully describe the immunosuppressive tumor microenvironment and identify biomarkers capable of predicting therapeutic resistance. An unsupervised clustering approach was utilized by Yang et al. to examine RNA-Seq data from 624 LUSC samples to identify patterns of gene expression in the tumor microenvironment. They identified expression patterns associated with T cell depletion, immunosuppressive cell activity, specific clinical characteristics, and patient responses to immunotherapeutic intervention; internal and external datasets were used to validate these results in the context of immune-depleted states. As a result, a new immunosuppressive subtype of LUSC was identified, which was distinguished by elevated PD-L1 expression and ICB resistance. This finding provides a strong foundation for further investigations into the underlying mechanisms of this resistance and the associated optimization of immunotherapeutic regimens (Yang ML et al., 2022). Gao et al. employed RNA-Seq data from TCGA and Gene Expression Omnibus (GEO) microarray datasets to ultimately discover five key genes associated with the formation and progression of LUSC. These genes are all involved in the dysregulation of the cell cycle, making them potential targets for therapeutic intervention. Survival analyses and Cox proportional risk models were used to include these five genes in a prognostic gene signature. This gene signature can predict patient overall survival and provides a basis for future research on the causes and treatment of LUSC, pending further experimental validation (Gao et al., 2020). Stewart et al. employed mass spectrometry-based proteomic data along with parallel mRNA and DNA analyses to aid in the categorization of LUSC at the molecular level and to identify the main factors responsible for these tumors, ultimately producing a proteomic map unifying known genomic, transcriptomic, and proteomic alterations. These integrated analyses provide an opportunity to identify therapeutic vulnerabilities that can be effectively treated. For instance, these findings indicate that immune combination therapies that target metabolic vulnerabilities and tumor-associated neutrophils may be effective against NFE2L2/KEAP1-mutated SCC tumors of the inflammatory or redox subtypes (Stewart et al., 2019). Yang et al. used data from 501 LUSC patients in the TCGA database to develop a model for predicting LUSC patient immunotherapy responses based on tumor-infiltrating immune cells (TIICs) within the tumor microenvironment. Single-sample gene set enrichment studies were employed to evaluate the level of TIIC, by grouping patients using NMF. The researchers then validated the accuracy of their model using two datasets (GSE126044 and GSE135222), by developing models using the least absolute contraction and selection operator (LASSO) regression screening and immunophenotyping score (IPTS) values. The prognostic performance of these scores was assessed in an independent cohort of LUSC patients using survival and ROC curve analyses. Furthermore, the underlying mechanisms of the identified IPTS molecular subtypes were investigated through enrichment and correlation analyses to predict the response of LUSC to immunotherapy. The use of TIIC-based IPTS molecular typing shows great potential for predicting the efficacy of immunotherapy and the prognosis of patients (Yang LG et al., 2022).

**FIGURE 6:**
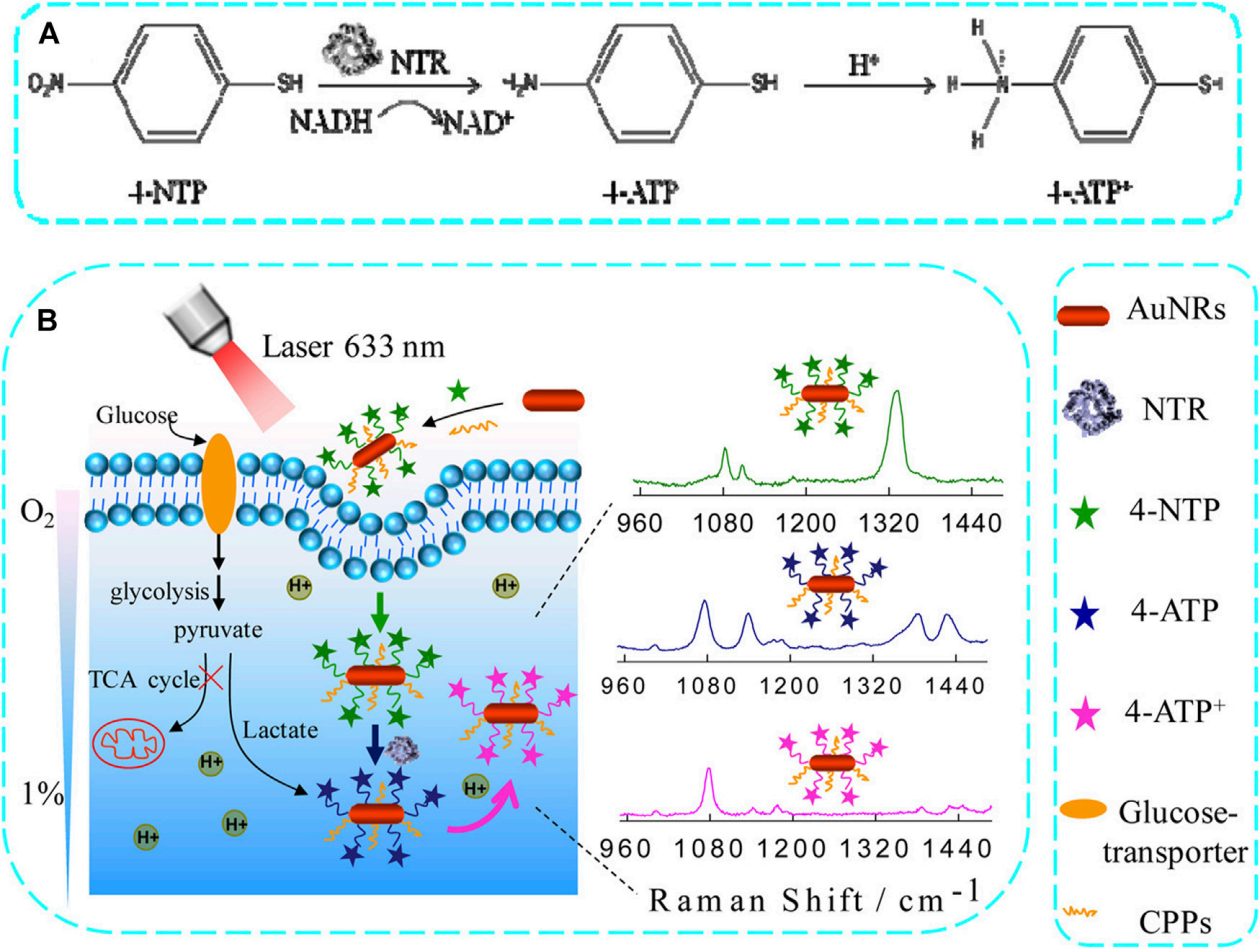
The use of an activated SERS nanoprobe for the quantitative assessment of hypoxia-induced lung tumor cell acidification. **(A)** NTR catalyzes the conversion of 4-NTP to 4-ATP using NADH. **(B)** A schematic overview of the application of AuNR@4 NTP@CPP SERS nanoprobes for the monitoring of hypoxia-induced acidification within cells (Ma et al., 2016).

Raman spectroscopy is well-suited for evaluating biomedical samples in a minimally invasive and accurate manner, allowing for effective identification of their molecular properties. Zhang et al. effectively used Raman microspectroscopy to detect the squamous cell carcinoma (SCC)-associated cancer field effect. Briefly, they employed Raman spectroscopy to analyze hematoxylin and eosin-stained tumor images and identify biochemical changes associated with SCC invasion and metastasis. These alterations include changes in lipids, DNA, and collagen in both normal and cancerous lesions. Using this method, they discovered that cancerous lesions exhibited substantial decreases in collagen-related Raman peak intensities (853, 936, and 1,248 cm^-1^), while peak intensities associated with DNA (720, 1,327 cm^-1^) and lipids (1,305 cm^-1^) were significantly increased. Consistent changes in peak intensity were observed in two different grades of SCC, providing valuable information about the cancer field effect associated with this type of cancer, as well as serving as a useful reference for the Raman-based diagnosis of SCCs (Zhang et al., 2019). Although ICB techniques have shown considerable potential for treating SCC, only a few patients achieve substantial benefits from this treatment, while others may develop serious irAEs. Therefore, it is imperative to develop the ability to identify patients who are most likely to benefit from ICB treatment, to improve patient outcomes and minimize patient risk. To achieve this objective, researchers in one study developed a SERS assay to examine CTCs at baseline and in response to stimulation with interferon (IFN)-γ. Through simultaneous integrated and single-cell analyses of these CTs, the researchers were able to conduct SERS assays capable of revealing the heterogeneous nature of tumors and provided valuable information for a thorough analysis of CTCs, which can influence clinical decision-making. To achieve this, anisotropic gold and silver alloy nanoboxes were implemented as SERS plasma substrates to enhance the signals associated with biomarkers of interest on CTC surfaces. The researchers were able to successfully distinguish between CTCs derived from melanoma cell lines of three separate patients using a unique four-surface marker CTC marker method. Among a group of 14 patients with melanoma who received anti-PD-1 treatment, the CTC signatures triggered by IFN-γ stimulation were able to accurately predict CTC changes in therapeutic responders. SERS-based detection methods can assist in identifying suitable patients who are highly eligible for ICB treatment (Li et al., 2022). Another investigation (Figure 6) demonstrated the development of an innovative activated SERS nanoprobe that can detect changes in pH caused by hypoxia. The SERS spectrum of gold nanorods functionalized with 4-nitrophenthiophenol (4-NTP) (AuNR@4-NTP) was observed in the presence of nitro reductase (NTR)-mediated hypoxia-induced reduction, which generated pH-sensitive 4-ATP. This reaction facilitated the efficient monitoring of acidification in live tissues and cells using SERS under hypoxic conditions. Dynamic pH analyses demonstrated that a decrease in pH from 7.1 to 6.5, caused by hypoxia, led to a corresponding increase in glycolytic activity, which was directly proportional to the level of hypoxia ranging from 15% to 1%. These results highlight the significant potential of SERS as a method for investigating hypoxia-related pathophysiological pathways, due to its beneficial sensing properties. SERS nanoprobe-based Raman spectroscopy can thus provide valuable insights for preclinical research, cancer treatment, and the personalized assessment of treatment outcomes (Ma et al., 2016). The feasibility of measuring the pH of lung cancer tissue under varying hypoxic conditions was examined by Ma et al. Under 20% O_2_ normoxic and 20% O_2_ hypoxic conditions, the tissue sections were cultured using AuNRs@4-NTP@CPPs. Following 3 hours of culture with AuNRs@4-NTP@CPPs under both typical (1% O_2_) and normal (20% O_2_) hypoxic conditions, SERS peaks were measured. SERS peaks were measured on tissue slices. After that, it was introduced to a pH 7.5 external medium. Sections treated at normal oxygen concentrations were used as controls, as shown in Figure 6A. As the oxygen content decreased from 20% to 1%, the intensity of the SERS peak at 1,438 cm^-1^ increased, whereas the intensity of the SERS peak at 1,069 cm^-1^ decreased. The intensity of the SERS peak exhibited an increase, although the peak intensity at 1,069 cm^-1^ remained relatively unchanged. Furthermore, lung cancer tissue sections were subjected to treatment with glucose and 2-DG, respectively. The Raman spectrum at 1,438 cm^-1^ indicated a slight increase in the SERS signal, while the SERS signal at 1,438 cm^-1^ was dramatically decreased in the glucose treatment group. The intensity of the SERS signal at 1,438 cm^-1^ was markedly reduced. Z-scan confocal superposition mapping imaging further demonstrates that SERS signals can remain within 500 microns. The experimental findings demonstrated that the SERS nanoprobes suggested by the researchers could provide high-contrast SERS imaging of pH values in deep tumor tissues under hypoxic conditions. A novel SERS nanoprobe was proposed to quantitatively detect the dynamic changes in intracellular acidification in living cells and tissues at various oxygen concentrations. SERS activity and NTR responsiveness were incorporated into the nanoprobe through the functionalization of 4-NTP at AuNRs. When 4-NTP is exposed to NTR, the nitro group of 4-NTP will undergo conversion into an amino group, causing a modification in the SERS spectrum. Therefore, the nanoprobe can be utilized under hypoxic conditions. Due to its pH sensitivity, the produced 4-ATP can be influenced by the changes in pH value in living cells and tissues, which in turn affects the SERS spectrum. The results of the dynamic pH analysis revealed an uneven decrease in pH as the oxygen content in living cells decreased. Specifically, it was also established that SERS nanoprobes can provide good quantitative measurement of local pH in lung cancer. The quantitative monitoring of the inhibitory effect of 2-DG and glucose on local pH and the promoting effect of glucose on glycolysis in lung cancer cells and tissues was conducted. The promoting effect on glycolysis was observed specifically under hypoxic conditions. Together, the researchers hope this will provide a potentially rich opportunity to understand the mechanisms of glycolysis under hypoxic conditions. To further the screening and optimization of anti-tumor drugs, it is important to comprehend the mechanism of glycolysis under hypoxic conditions (Ma et al., 2016).

**FIGURE 7:**
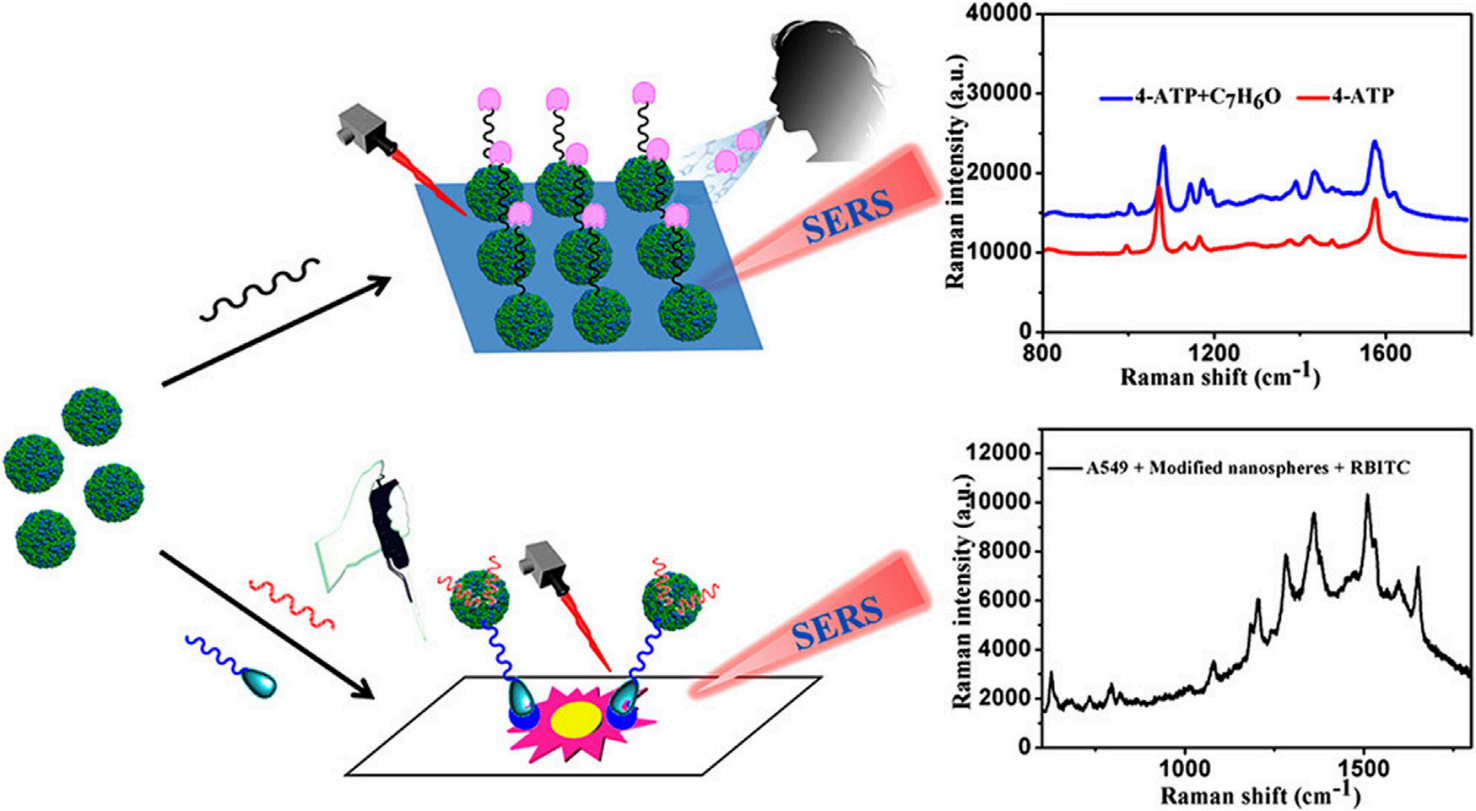
The application of photochemically synthesized heterostructured porous copper FeSe_2_/Au nanospheres as SERS sensors to aid in the ultrasensitive detection of lung cancer cells are associated biomarkers of interest (Wen et al., 2019).

Andreou et al. described a comprehensive imaging technique that used SERS nanoprobes and machine learning to assess the early efficacy of ICB in a murine tumor model system. Using antibody-functionalized SERS nanoprobes, they simultaneously observed eight targets relevant to this immunotherapeutic approach. This resulted in the generation of multiplexed images which underwent spectral resolution and spatial segmentation into superpixels according to the unmixed signal. The aforementioned superpixels were subsequently used in the training of a machine-learning model. The model that emerged from this process effectively distinguished between treated and untreated mice, as well as identified specific regions of tumors that exhibited differential responses to the treatment. This strategy thus holds promise as a means of predicting whether tumors will respond to particular interventions, providing a potential means of better addressing therapeutic resistance and tumor variability and treatment resistance (Andreou et al., 2022). In another study, researchers designed novel renewable FeSe_2_/Au nanospheres with multistage porosity (Figure 7) and effectively used them as tools to sensitively detect aldehydes and lung cancer cell biomarkers (Wen et al., 2019). To produce these nanospheres, photoreduction was performed on CuFeSe_2_ to generate Au shells, and the resulting heterogeneous nanosphere framework was loaded with 4-ATP, which was selected as a Raman active probe. Subsequently, gaseous aldehyde molecules were sensitized to these nanospheres via the formation of a C=N bond, resulting in a detection limit of 1.0 ppb. The SERS activity of the prepared folic acid-coupled nanospheres against rhodamine isothiocyanate B was also exceptionally high, enabling the sensitive and specific identification of A549 lung tumor cells. The developed synthetic renewable copper FeSe_2_/Au heterostructured nanospheres thus show significant potential as a versatile tool for many applications in medicinal, biotechnological, and environmental research fields. Using the SERS approach, tumor biomarkers or embryonic cancer cells can be quickly and sensitively identified. The use of hot spots in one- or two-dimensional geometry is less effective because of the poor affinity of the molecules for the metal surface, the complexity of the sample, and the ineffective use of hot spots in single or multiple hot spots. To detect lung cancer cells and biomarkers with high specificity and sensitivity, Wen et al. introduced new renewable CuFeSe_2_/Au heterostructured nanospheres with layered porosity. Photoreduction was used to produce heterostructured nanospheres by depositing gold shells onto CuFeSe_2_ frames. The Raman active probe molecule 4-ATP was first grafted onto the CuFeSe_2_ framework. CuFeSe_2_/Au nanospheres were grafted first, followed by the bonding of gaseous aldehyde molecules to C=N on the nanospheres, with a detection limit of 1.0 ppb. Moreover, the produced nanospheres conjugated with folate (FA) exhibited strong SERS activity against Rhodamine B isothiocyanate (RBITC), suggesting that they could be employed for the sensitive and targeted detection of A549 cells. The results of our study indicate that synthetic renewable CuFeSe_2_/Au heterostructured nanospheres have the potential to be used in several fields such as medicine, biotechnology, and environmental science. The fabrication of CuFeSe2/Au nanospheres involves a three-step synthesis process. Initially, CuFeSe_2_ was synthesized using thermal decomposition, followed by the preparation of NCs using the same method. The TEM images of CuFeSe_2_ NCs revealed a uniform and homogeneous spherical morphology with a diameter of 5.7 nm. The HRTEM images revealed a D-spacing of 0.272 nm, which corresponds to the (200) crystallographic plane of CuFeSe_2_ in the tetragonal phase. The XRD investigation used Cu Kα radiation with a wavelength of 1.5418 Å. The analysis of all samples was conducted using PHI-5702 XPS. The process of optical reduction was carried out using a xenon lamp of the model HX-F300. Infrared spectra were obtained using a Fourier Transform Infrared (FT-IR) spectrometer (Bruker Vertex 70) fitted with a single-reflector ATR attachment. The chemical composition of nanostructures was determined using Inductively Coupled Plasma Atomic Emission Spectroscopy (ICP-AES). The CuFeSe_2_/Au nanospheres use numerous surface holes on the nanostructure to overcome the long-term limitation of gas molecule absorption on a solid substrate. As a result, the detection limit is reduced to 1.0 ppb when 4-ATP molecules with Raman activity are pre-adsorbed to CuFeSe_2_/Au nanospheres, thereby significantly enhancing the sensitivity of gaseous aldehydes. Simultaneously, CuFeSe_2_/Au nanospheres exhibited a high SERS activity toward RBITC, resulting in increased selectivity and sensitivity of the nanostructures towards A549 cells. Furthermore, the heterostructured nanospheres showed remarkable photocatalytic cleaning properties and efficient renewable properties. This innovative renewable porous CuFeSe_2_/Au nanosphere is expected to have significant potential for applications in sensing, biomedical, and bioanalysis. Wen et al. (2019).

## Conclusion and prospects

The blockade of the PD-1/PD-L1 pathway has transformed the treatment of advanced non-small cell lung cancer and significantly improved the overall survival of patients (Linehan and Forde, 2020). An increasing number of clinical trials have investigated the potential roles of neoadjuvant or adjuvant ICB treatment in patients with early-stage lung cancer. Although TMB and PD-L1 expression levels are commonly used for patient selection, they are not excellent biomarkers. Due to the complex interactions among tumors, immune cells, and the surrounding microenvironment, it is crucial to develop multidimensional immune profiles that incorporate a series of complementary predictive biomarkers to facilitate individualized immunotherapeutic treatment (Huang and Yang, 2021). Raman spectroscopy is an effective analytical method that can be employed to determine the chemical composition of various biological targets, such as tissues, cells, and biofluid samples. By leveraging the differences between normal and malignant tissues, Raman spectroscopy can enable the accurate, noninvasive, and rapid detection of tissue molecular composition. This method is well-suited for deep edge assessment without the need for exogenous drug application due to its high level of specificity and ability to characterize tissues beyond mucous membranes. Raman spectroscopy can detect biochemical changes in tissues and cells that are associated with disease development. This technique is valuable because it can identify these changes at the molecular level, enabling the diagnosis, prognosis, and evaluation of treatments for patients. The combination of FTIR spectroscopy and Raman spectroscopy can enhance diagnostic attempts for lung cancer. SERS-based methods can aid in the biochemical analysis of serum for intraoperative pathological diagnosis, as well as enable the detection of ICB. CNN approaches using Raman spectrograms with two Villaman spectrograms can effectively categorize cancer-related tissues. Additionally, laser tweezer Raman spectroscopy enables drug analysis at the cellular level.

The Raman spectral speed is comparatively slow, which significantly limits the clinical implementation of this technology. Therefore, there is still a need for additional efforts to assist in the translation of Raman spectroscopy into clinical healthcare settings. This work should be based on the findings of well-designed randomized clinical trials, which show that this technique has better clinical outcomes and is more cost-effective than other strategies currently used in practice. While the application of Raman spectroscopy in clinical diagnosis and treatment holds promise, it also faces several limitations. For instance, the tissue Raman signal tends to be weak, and the acquisition speed of spectra is relatively slow. Moreover, the overlap of various vibration peaks and susceptibility of Raman scattering intensity to optical system parameters pose challenges. In addition, fluorescence interference complicates the study of Fourier transform Raman spectra, while nonlinear curve issues frequently develop in Fourier transform spectral analysis. Raman spectroscopy molecular imaging combines imaging technology, molecular biology, information processing technology, chemistry, and medical statistics to achieve real-time, non-invasive, dynamic, and *in vivo* imaging at the molecular level. It offers several advantages, including high resolution, non-radioactivity, easy detection, and low cost. This technique can be used to observe tumor-targeting markers in living cells. The occurrence, development, and metastasis process of tumors in small animals, the safety assessment of drugs and pre-clinical pharmacological research *in vivo*, as well as the detection and treatment of endoscopy, have exceeded the constraints of conventional imaging technology, which only depict changes in tissue structure, and have emerged as prominent areas of research in the field of molecular imaging. These areas possess significant potential for practical implementation in the field of medical imaging (Schuster et al., 2000; Enami et al., 2007; Johannessen et al., 2007).
